# *Dalbergia odorifera* undergoes massive molecular shifts in response to waterlogging combined with salinity

**DOI:** 10.1093/plphys/kiad639

**Published:** 2023-12-04

**Authors:** El-Hadji Malick Cisse, Bai-Hui Jiang, Li-Yan Yin, Ling-Feng Miao, Jing-Jing Zhou, Francine Ngaffo Mekontso, Da-Dong Li, Li-Shan Xiang, Fan Yang

**Affiliations:** Key Laboratory of Agro-Forestry Environmental Processes and Ecological Regulation of Hainan Province, Center for Eco-Environment Restoration Engineering of Hainan Province, School of Ecological and Environmental Sciences, Hainan University, Haikou 570228, China; School of Life Sciences, Hainan University, Haikou 570228, China; Key Laboratory of Agro-Forestry Environmental Processes and Ecological Regulation of Hainan Province, Center for Eco-Environment Restoration Engineering of Hainan Province, School of Ecological and Environmental Sciences, Hainan University, Haikou 570228, China; School of Life Sciences, Hainan University, Haikou 570228, China; Key Laboratory of Agro-Forestry Environmental Processes and Ecological Regulation of Hainan Province, Center for Eco-Environment Restoration Engineering of Hainan Province, School of Ecological and Environmental Sciences, Hainan University, Haikou 570228, China; School of Plant Protection, Hainan University, Haikou 570228, China; Key Laboratory of Agro-Forestry Environmental Processes and Ecological Regulation of Hainan Province, Center for Eco-Environment Restoration Engineering of Hainan Province, School of Ecological and Environmental Sciences, Hainan University, Haikou 570228, China; School of Food Science and Engineering, Hainan University, Haikou 570228, China; Key Laboratory of Agro-Forestry Environmental Processes and Ecological Regulation of Hainan Province, Center for Eco-Environment Restoration Engineering of Hainan Province, School of Ecological and Environmental Sciences, Hainan University, Haikou 570228, China; School of Life Sciences, Hainan University, Haikou 570228, China; Key Laboratory of Agro-Forestry Environmental Processes and Ecological Regulation of Hainan Province, Center for Eco-Environment Restoration Engineering of Hainan Province, School of Ecological and Environmental Sciences, Hainan University, Haikou 570228, China; Key Laboratory of Agro-Forestry Environmental Processes and Ecological Regulation of Hainan Province, Center for Eco-Environment Restoration Engineering of Hainan Province, School of Ecological and Environmental Sciences, Hainan University, Haikou 570228, China

## Abstract

Field and greenhouse studies attempting to describe the molecular responses of plant species under waterlogging (WL) combined with salinity (ST) are almost nonexistent. We integrated transcriptional, metabolic, and physiological responses involving several crucial transcripts and common differentially expressed genes and metabolites in fragrant rosewood (*Dalbergia odorifera*) leaflets to dissect plant-specific molecular responses and patterns under WL combined with ST (SWL). We discovered that the synergistic pattern of the transcriptional response of fragrant rosewood under SWL was exclusively characterized by the number of regulated transcripts. The response patterns under SWL based on transcriptome and metabolome regulation statuses revealed different patterns (additive, dominant, neutral, minor, unilateral, and antagonistic) of transcripts or metabolites that were commonly regulated or expressed uniquely under SWL. Under SWL, the synergistic transcriptional response of several functional gene subsets was positively associated with several metabolomic and physiological responses related to the shutdown of the photosynthetic apparatus and the extensive degradation of starch into saccharides through α-amylase, β-amylase, and α-glucosidase or plastoglobuli accumulation. The dissimilarity between the regulation status and number of transcripts in plants under combined stresses led to nonsynergistic responses in several physiological and phytohormonal traits. As inferred from the impressive synergistic transcriptional response to morpho-physiological changes, combined stresses exhibited a gradually decreasing effect on the changes observed at the molecular level compared to those in the morphological one. Here, by characterizing the molecular responses and patterns of plant species under SWL, our study considerably improves our understanding of the molecular mechanisms underlying combined stress.

## Teaser

The molecular responses to waterlogging combined with salinity correlated with plant morpho-physiology and anatomy shed light on the interplay between gene regulation and pattern, metabolites, and plant physiology.

## Introduction

**“**The road is long, and the time is short, so we better be in a hurry,” was an acknowledgment by Zandalinas and Mittler (2022) in a recent report apropos of the lack of data involving the multifactorial effects of combined stresses on plant growth and development. Global climate change implies an increase in extreme environmental events that negatively affect forest tree species distribution and development. Natural catastrophes, such as typhoons and storms, are making their signature in coastal and wetland areas, influencing ecosystem resistance and resilience (Patrick et al. 2022). Trees are a main factor sustaining ecological restoration and management under hazardous flood conditions and will ipso facto increase ecosystem resilience (Nilsson et al. 2018). Marcar et al. (2002) established that commercial seeds, seedlings, or tree species depend on multifactorial stress tolerance particularly in the face of increasing events related to climate change. Here comes advocacy about the scarcity of molecular studies tree species compared to crops. Sustain food security through comprehensive research focusing on molecular profiles and pathways in crops under harsh conditions are imperative objectives to achieve. But, can it be imagined to reach that objective without an equal understanding of the molecular responses of the species that sustain most terrestrial ecosystems? Many landscapes throughout the world are exposed to synchronized waterlogging (WL) and salinity (ST). The simultaneous occurrence of WL and ST in landscapes, such as semiarid regions, is increasing (Carter et al. 2006; Duhan et al. 2019). A previous study on crops highlighted that the molecular response of plants to a combination of stresses is unequal to that to each stress applied individually (Mittler 2006). However, little is known about the molecular mechanisms underlying the responses of plant species, particularly forest trees, to a combination of different stresses (Sewelam et al. 2020; Zandalinas et al. 2020). We do not know what transcriptional and metabolomic variations tree species exhibit to defend themselves against a combination of WL and SL conditions. The limited pieces of information regarding the responses of trees to a combination of WL and ST (SWL) concern plant physiology, metabolites, ions, and organic solute variation in different plant organs (leaf and/or root) (Barrett-Lennard and Shabala 2013; Behr et al. 2017).

Moreover, only a few studies, such as the work of Shaar-Moshe et al. (2017), have attempted to describe the common transcription patterns of plants under combined stresses. The characterization of the shared metabolites and gene patterns during combined stresses will considerably contribute to our understanding of the uniqueness of plant responses under combined stresses and provide several markers of these responses. Transcripts and metabolites that can be crucial for plant breeding and genetic engineering for stress tolerance are expressed under ST, WL, and SWL. The knowledge of their pattern is the key element that will help find markers or candidates of stress-responsive genes. The transcriptomic analysis of various plant species under combined stresses revealed a mosaic of transcript behavior. Transcriptomic profiles cannot be predicted from the related single stressor (Prasch and Sonnewald 2013; Coolen et al. 2016; Cohen et al. 2021; Zandalinas et al. 2021). Combined stresses massively increase the level of differentially expressed and regulated genes as reflected by drastic changes in plant genomes and morphologies (Desaint et al. 2021). The integration of multiple signals in plants under combined stresses that leads to a systemic response is far from being taken as an ipse dixit. In Arabidopsis (*Arabidopsis thaliana*), heat stress associated with high light generated systemic signals linked to reactive oxygen species (ROS) that resulted in a systemic response (Zandalinas et al. 2020).

Most of the studies cited above are related to the model plant *Arabidopsis* and to the combination of ST, heat stress, high light, or drought. The genetic manipulation and use in a greenhouse or experimental chamber of *Arabidopsis* are more convenient than those of woody plants. A previous study focusing on the tolerance of different wetland tree species showed the crucial role of ion uptake regulation under SWL (Carter et al. 2006). However, comprehensive transcriptomic analysis integrated with metabolic and physiological analyses in tree-specific organs is needed to understand how trees face combined stresses. Here, we investigated the metabolomic and transcriptional patterns of the non-halophyte forest species fragrant rosewood (*Dalbergia odorifera*) T. Chen. Fragrant rosewood possesses WL and ST tolerance and could be used as a preferred tree species in the upper parts of the intertidal zones periodically subjected to combined WL and salt stresses. This study investigated the molecular responses (transcriptomic integrated with metabolomic) and patterns of plants under WL combined with ST. Six woody plant species, including fragrant rosewood, have been suggested to be suitable for the ecological restoration of flooded watershed areas (Ma et al. 2019). Fragrant rosewood exhibits new leaf growth and high fresh biomass growth rate under long-term hydro-culture. It belongs to the Fabaceae family and has a high concentration of phenolics in its organs, thus possessing impressive antioxidant activity. Most flood-related ecosystem restoration projects aim to increase biodiversity by adding exotic woody plants, and recent initiatives have included climate change adaptation (Nilsson et al. 2018). Tree species selected on the basis of tolerance to combined stresses have been established to be more likely to perform better in stressful areas than those tolerant of a single abiotic stress (Marcar et al. 2002). Here, we reveal the unique metabolomic, transcriptomic, physiological, and hormonal responses of fragrant rosewood to a combination of WL and ST conditions. Although fragrant rosewood possesses certain salt tolerance and can grow well under WL, we hypothesize that SWL induces massive shutdown of the photosynthesis, starch formation, and biomass accumulation in this forest species. Moreover, fragrant rosewood seedlings under SWL can be plausibly expected to have the same specific pattern modes in terms of metabolome and transcriptome profiles. Given that most early studies focusing on combined stresses were associated with plant acclimation, the non-acclimation stress (SWL) applied in the present study would provide important insights into plant survival and adaptation responses under combined stresses.

## Results

### Primary outcome of SWL in plants: a remarkable and extensive enhancement in the expression of genes and metabolites, surpassing anything observed under a single stress

Metabolomic characterization under combined and single stresses showed the uniqueness of the metabolome profile of fragrant rosewood (*D. odorifera*). Metabolomic clustering analysis (Supplemental Fig. S1, A to C) revealed major differences between metabolite levels under control, single, and combined stress treatments. The first component (PC1) of principal component analysis (PCA) separated control (CT) and SWL, whereas the second component (PC2) separated CT and WL. Among the 3 independent biological samples under ST, PC1 separated CT and 2 groups of ST combined with 3 groups of SWL (Supplemental Fig. S1B). UPLC‒MS/MS detected 572 metabolites under each treatment and 137 DMs under all treatments. Differential gene expression analysis was performed by using DESeq2 to further characterize the molecular responses of plants under SWL. The results showed that an impressive number of regulated transcripts were involved under SWL (14053) relative to under WL (4111) or ST (2670). The comparison of all 3 treatments with CT revealed that the downregulated DEGs outnumbered upregulated transcripts (Supplemental Table S1). The intersecting sections of the Venn diagrams for the DMs revealed various clusters showing shared metabolites among combined stresses and single treatments (Supplemental Fig. S1, D to F). The hierarchical clustering of the most highly and weakly expressed genes in fragrant rosewood leaflet samples determined with RNA-seq showed that the patterns of clusters from biological replicates under ST, WL, or CT were different from those under combined stresses (Supplemental Fig. S2, A to C). These results revealed that transcripts with identical or similar expression patterns under different treatments clustered into classes and that these similar transcripts could be regrouped for further analysis. The overlapping area of the Venn diagrams (Supplemental Fig. S2, D to F) of the DEGs resulted in different groups that indicated intersection between combined stresses and other treatments. This step is crucial for isolating the commonly shared genes among WL, ST, and SWL. Volcano and MA plots (Supplemental Fig. S3) were used to represent the synergistic effects on the number of transcripts of combined stresses in plants compared with those of single stresses. DEGs whether either up- or downregulated showed massive abundance under combined stresses. The mobilization herculean of these genes under combined stresses is a fundamental response of plants for tolerance and survival under combined stresses. Weighted gene co-expression network analysis (WGCNA) (Supplemental Figs. S4 to S7) showed that similarly expressed genes in each biological replicate under SWL were functionally associated in the blue module, suggesting that several genes are influenced only by SWL.

### SWL induced distinct patterns of commonly expressed genes and metabolites

**Figure 1.**
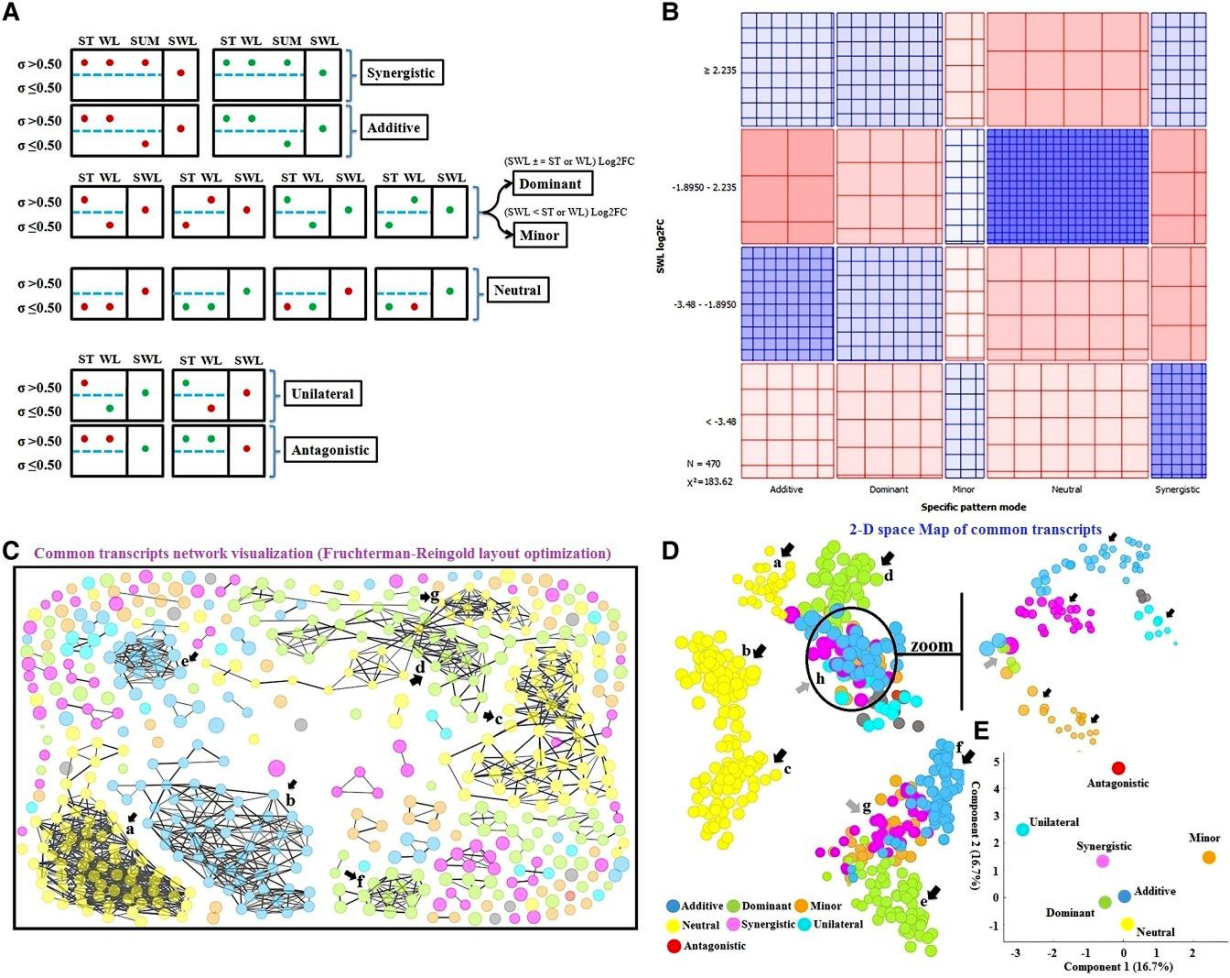
Metabolomic and transcriptional patterns based on log2FC, regulation status (down, green or up, red), and standard deviation (Sd ≤ 0.50) of fragrant rosewood responses to single and combined stresses. Illustrative schema of molecular expression (metabolites and genes) that showed 7 pattern modes (synergistic, additive, dominant, neutral, minor, unilateral, and antagonistic) found in *D. odorifera* under combined stress compared to each single stress **A)**. Two-way contingency diagram of the common genes pattern based on the pattern modes and log2FC in SWL-treated seedlings, the difference between observed and expected frequencies which is proportional to the standard Pearson residual appears as the density of shading, using color to indicate whether the deviation from independence is positive (blue) or negative (red) **B)**. Two-dimensional common genes projection; genes network explorer based on Fruchterman–Reingold layout optimization **C)** and t-distributed stochastic neighbor embedding **D)**. Correspondence analysis for categorical multivariate transcriptomic data in the observed pattern in SWL **E)**. The pattern modes were represented as follows: synergistic (pink), additive (blue), dominant (green), neutral (yellow), minor (orange), unilateral (light blue), and antagonistic (red).

Focusing on commonly expressed transcripts and determining their patterns is essential for effectively searching for a marker or candidate gene with high efficiency under single and combined stresses. Understanding the patterns of these transcripts and/or metabolites is a pivotal element in identifying markers or candidates for stress-responsive genes. The common metabolite and transcriptional pattern under combined stresses based on each molecular constituent under a single stress and the Sd revealed 7 patterns involving synergistic, additive, dominant, neutral, minor, unilateral, and antagonistic modes (Fig. 1A; Supplemental Fig. S7; Supplemental Tables S2 and S3). The neutral pattern predominated in metabolomic and transcriptomic profiles. A total of 7.38% of the metabolites presented the antagonistic mode in which they were oppositely regulated under single and combined stresses. The well-known synergistic pattern of combined stresses in plants does not apply to the commonly expressed metabolites in fragrant rosewood leaflets under SWL. The rest of the assigned metabolites were distributed in the dominant (5.80%), minor (6.68%), and unilateral (11.07%) modes. Common DMs were selected based on variable importance in projection (VIP) (VIP ≥ 1) and fold change (FC) ≥ 2 (upregulated metabolites) or FC ≤ 0.5 (downregulated metabolites) to identify a metabolic pattern under SWL with increased informativeness. A total of 32 DMs were found, most of which were in the neutral mode (50%). Of the DMs, 25.01% were in the synergistic and additive modes, and the rest were distributed in the minor mode (Supplemental Table S2). Interestingly, the percentage of common DEGs distributed in the synergistic and additive modes (31.03%) was approximately the same as that in the neutral mode (31.98%). These results indicated that numerous DEGs were massively up- or downregulated under SWL relative to under ST or WL (Supplemental Fig. S7D). The numbers and profiles of common DEGs were quite different from those of DMs or the overall metabolites. However, a high number of commonly regulated DMs or DEGs were expressed similarly in fragrant rosewood leaflets under combined or single stresses.

### Unraveling the interplay of common gene expression and metabolite pattern profiles under combined stresses through clustering and network analysis

**Figure 2.**
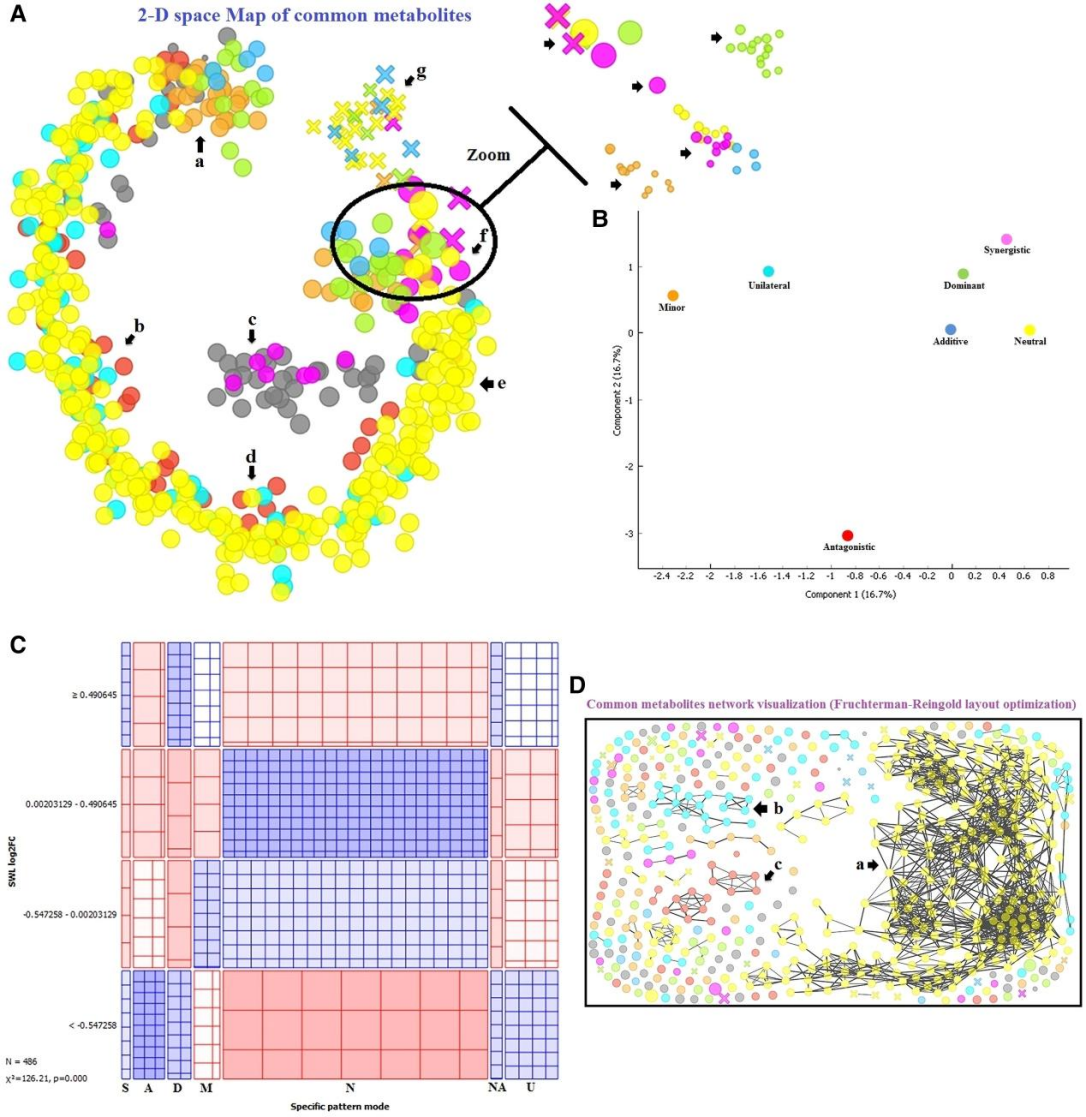
Integrated metabolomic analysis and visualization: exploring patterns and networks in fragrant rosewood under single and combined stresses. Common metabolites network explorer based on Fruchterman–Reingold layout optimization **A)**. Correspondence analysis for categorical multivariate metabolomic data in the observed pattern in SWL **B)**. Two-way contingency diagram of the common metabolites pattern based on the pattern modes and log2FC in SWL-treated seedlings **C)**. Common metabolome network explorer based on Fruchterman–Reingold layout optimization **D)**. The pattern modes were represented as follows: synergistic (S, pink), additive (A, blue), dominant (D, green), neutral (N, yellow), minor (M, orange), unilateral (U, light blue), antagonistic (A, red), and non-assigned (NA).

Figures 1B and 2C reveal a strong association between the pattern mode and SWL-log2FC of the common metabolites and transcripts. The observed frequencies in most quadrants exhibited significant deviations from the expected frequencies, indicating considerable differences. However, the synergistic pattern expected for the commonly expressed transcripts or metabolites was less pronounced than the other pattern modes. The global profile of several pattern modes of the common differential transcripts (Fig. 1, C and D) was differentially affected relative to that of the expressed metabolites (Fig. 2, A, B and D). The t-distributed stochastic neighbor embedding method (Figs. 1C and 2D) and multidimensional scaling maps (Figs. 1D and 2A) revealed a clustering structure in which several common genes and metabolites regulated under SWL in fragrant rosewood formed several populations in accordance with regulation status and size in SWL leaflets. The network of these potential SWL molecular markers showed several genes or metabolites that might play the same role in plants under SWL in accordance with their pattern mode. The commonly expressed metabolites were regrouped in several regions in which the common DMs clustered in zone a (Fig. 2A). Several DMs in zone f clustered with the rest of the metabolites that were distributed in zones a–e. The common transcripts regrouped on the basis of their pattern (a, b, c, d, e, and f) and size under SWL (log2FC) into 3 distinct gene clusters as shown in Fig. 1D despite having the same pattern mode (a, b, and c). Subpopulations can be clearly observed in the area wherein the common genes (h, Fig. 1D) or metabolites (f, Fig. 2A) were mixed, demonstrating how pattern modes and expression levels play crucial roles in the molecular network of several markers in plants under combined stresses.

### Shutdown of the photosynthetic apparatus by SWL through *LHCB* and *PSA* genes and massive plastoglobulus accumulation and starch degradation

**Figure 3.**
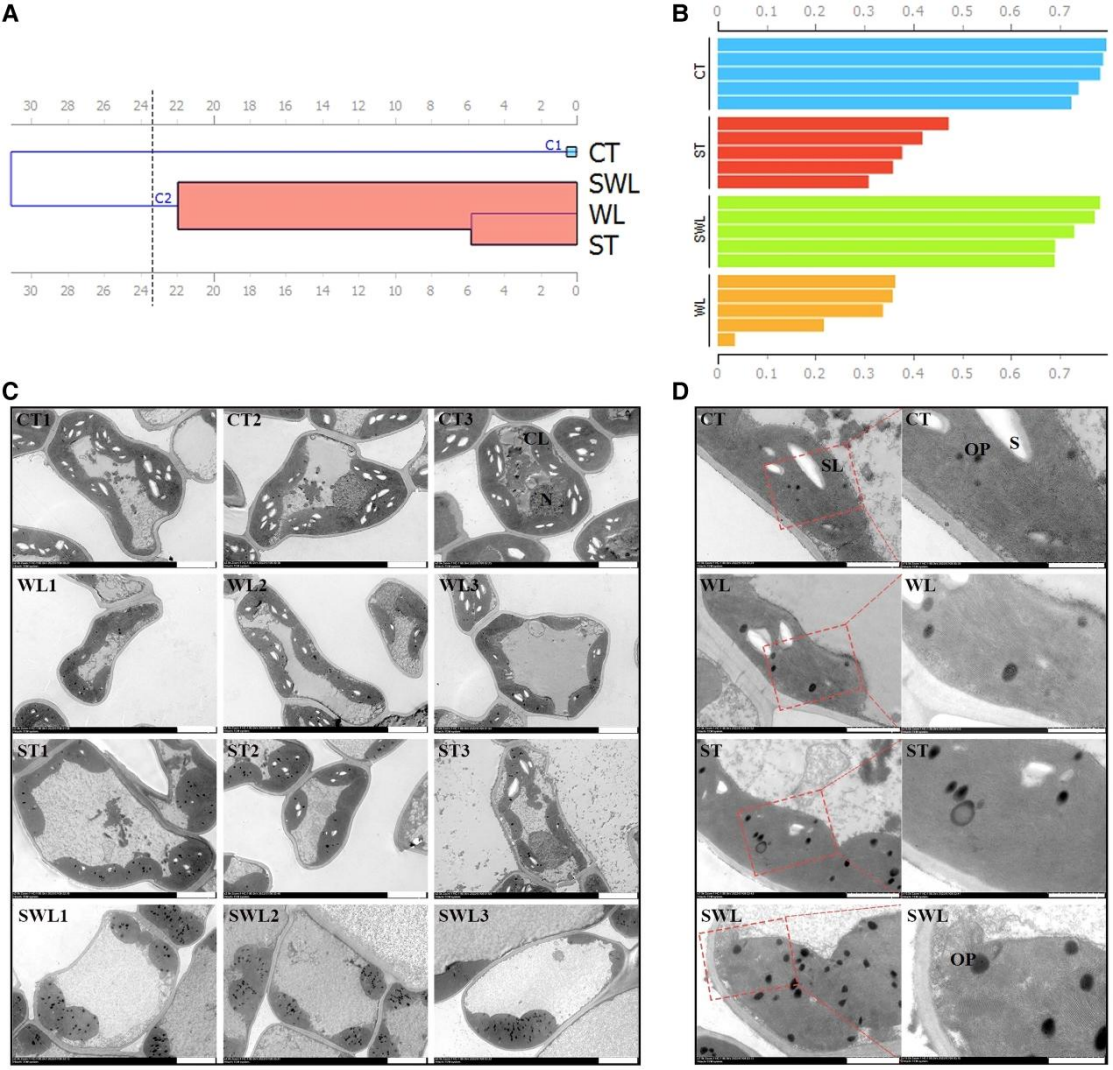
Comparative analysis of photosynthetic responses in fragrant rosewood to single and combined stresses. Dendrogram of hierarchical clustering of the dataset regrouping different photosynthetic related-parameters (glucose, sucrose, and fructose, starch, and number of starch granules, net photosynthetic rate, stomatal conductance, and ABA, GA_3_, and IAA) among CT, ST, WL, and SWL **A)** (C1 = cluster 1 and C2 = cluster 2). Silhouette analysis of the different clusters among different treatments based on Euclidean distance metric **B)**. Comparative images of chloroplast ultrastructure that showed starch and plastoglobuli variations among CT, ST, WL, and SWL: All the images of the chloroplast ultrastructure in the panel had a scale bar (bar in white) of 5 *μ*m **C)**, images of the chloroplast ultrastructure of CT1, ST1, WL1, and SWL1 **D)** at 2 *μ*m of scale bar showed at the left of panel **D** and 1 *μ*m corresponding to the images at the right of panel **D**. CL, chloroplast; N, nucleus; OP, osmiophilic plastoglobuli; S, starch granule; SL, stromal lamellae. Treatments are presented as follows: control (CT), salinity (ST), waterlogging (WL), and combination of WL and ST (SWL).

**Figure 4.**
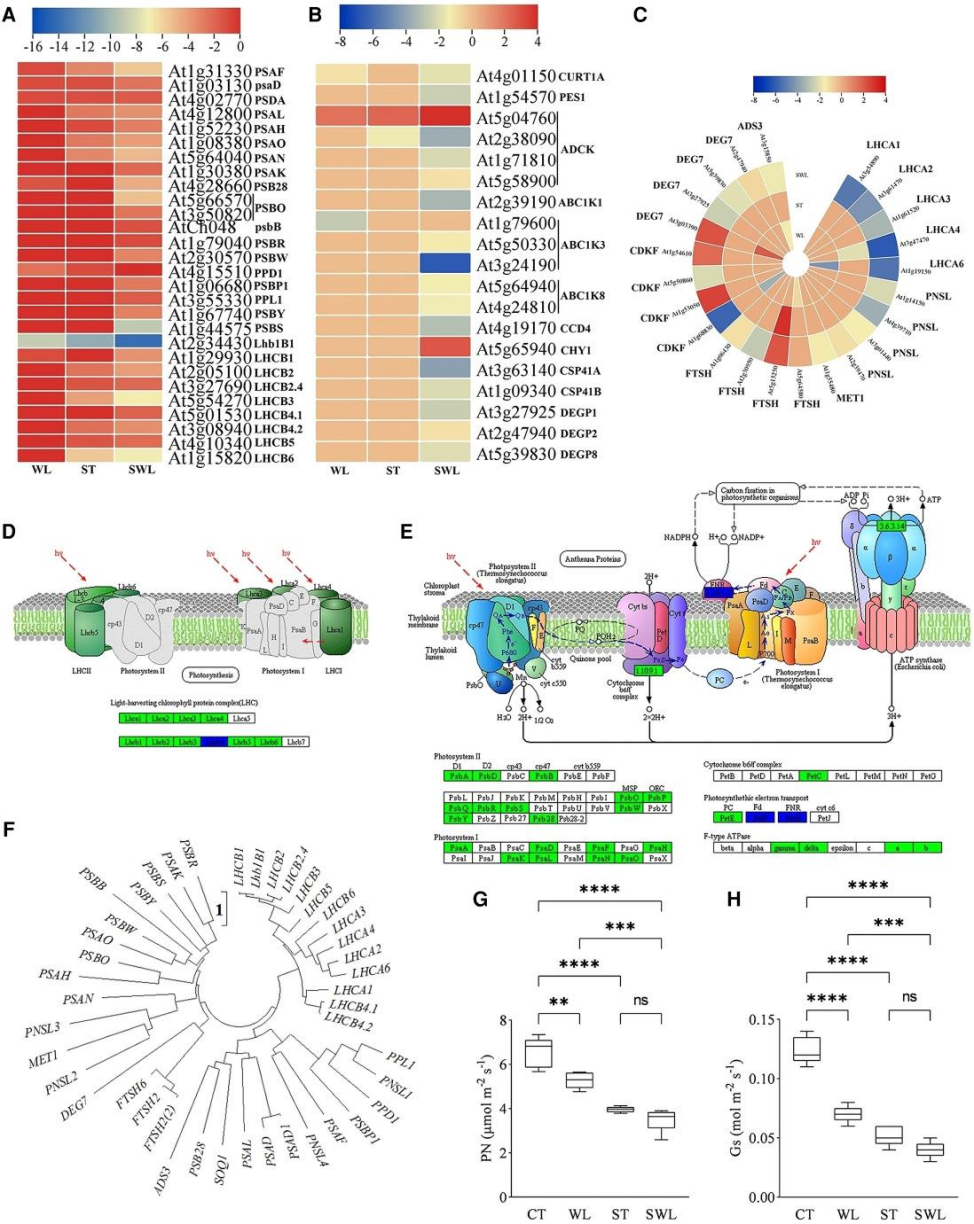
Transcriptomic and physiological analyses of the fragrant rosewood photosynthesis under single and combined stresses. Heat maps of photosynthetic related genes in photosystem I, photosystem II, and photosynthesis light harvesting (**A** and **B**), and plastoglobule modules **C)**. Schematic representation of the significantly enriched KEGG pathway of the photosynthesis-antenna proteins **D)** and photosynthetic apparatus **E)** under SWL that showed the regulation mode and relation of genes belonging to photosystems I and II, cytochrome b6/f complex, photosynthetic electron transport chain, and/or F-type ATPase; KO nodes containing only upregulated differential genes are marked in red, KO nodes containing only downregulated differential genes are marked in green, and KO nodes containing both upregulated and downregulated genes are marked in blue. Photosynthetic proteins-phylogeny tree based on the test Neighbour-Joining Tree and scale bar indicates number of changes per site **F)**. Variation of net photosynthetic (PN) rate **G)** and stomatal conductance (Gs) **H)** in fragrant rosewood under single and combined stresses. Treatments are presented as follows: control (CT), salinity (ST), waterlogging (WL), and combination of WL and ST (SWL). The signs such as *, **, ***, ****, and NS represented the significance difference among different treatments. *P < 0.05, **P < 0.01, ***P = 0.0001, ****P < 0.0001; NS, not significant. Data are expressed as means ± Sd (5 replicates from 5 different seedlings), and significant differences between means were determined at a *P*-value of ≤0.05 with 1-way ANOVA test. The perpendicular line within each box represents the median value and the ends of the box represent the 3rd and 1st quartile. The median is the value separating the higher half from the lower half of data sample. The lower edge of the box is Q1, which is the median of the lower half of the dataset. The upper edge is Q3, which is the median of the upper half of the dataset.

The photosynthetic dendrogram based on physiological indices showed 2 different clusters regrouping the CT and a second group containing ST, WL, and SWL (Fig. 3A). Figure 3B shows that the silhouette values under the CT and SWL treatments were close to 1 in contrast to those under ST or WL. The values found in the ST and WL clusters depicted 2 possibilities that included the proximity of the CT and/or SWL groups. However, Fig. 3A shows that both clusters were close to the SWL group. The chloroplast ultrastructure of fragrant rosewood leaflets was analyzed by using transmission microscopy to obtain an improved understanding of plant leaflet photosynthetic variations under combined stresses (Fig. 3, C and D). The density of the chloroplasts in the leaflet cells under CT was greater than that under single and combined stresses. The images showed in Fig. 3, C and D reflect the synergistic effects of combined stresses on plants, specifically, the reduction in starch granule size and number and the increase in osmiophilic plastoglobuli (OP) under SWL. The thylakoid-associated lipids in OP that actively participate in thylakoid function from biogenesis to senescence considerably accumulated in SWL-treated leaflets. The results revealed a massive synergistic shutdown in the photosynthetic apparatus that is reflected by the suppression of several light-harvesting complexes (I and II), chlorophyll a/b binding proteins, and genes related photosystems I and II (Fig. 4A and Supplemental Fig. S8). The same pattern was presented by the genes involved in photosystem II subunits and plastoglobule GO modules (Fig. 4, A to C and Supplemental Fig. S8E). The core subunit of photosystem PSA (D, F, H, L, N, and O) and outer light-harvesting complexes comprising the proteins LHCA (1, 2, and 4) and LHCB (1 to 5) related genes were strongly downregulated under SWL relative to under ST or WL. The frequency (ω) of *LHCB1* (16.33%), *LHCA2* (12.24%), and *LHCA4* (8.16%) was higher than that of the other genes related to light-harvesting complexes (Supplemental Fig. S8A). Figure 4, D and E depict the dramatic shutdown of the light-harvesting chlorophyll protein complex, photosystems I and II, and photosynthetic electron transport in fragrant rosewood leaflets under SWL. The same pattern was shown by genes related to plastoglobule biosynthesis, among which the activity of the abc1 complex (*ABC1*, a member of the protein kinase family) and aarF domain-containing kinase (*ADCK*) genes were predominant (Supplemental Fig. S8B). *ADCK* and *ABC1* expression variations are crucial for photosynthetic performance and plant stress tolerance. The transcript involved in photosynthesis that was massively regulated under SWL that might have a common ancestor is shown in Fig. 4F. The transcriptional changes in fragrant rosewood leaflets described above resulted in a significant decrease in net photosynthetic rate and stomatal conductance under SWL relative to under the control and WL (Fig. 4, G and H).

### Diametrical or similar regulation patterns of synergistically expressed genes caused by combined stresses drove the nonsynergistic or synergistic response of ABA and saccharide accumulation

**Figure 5.**
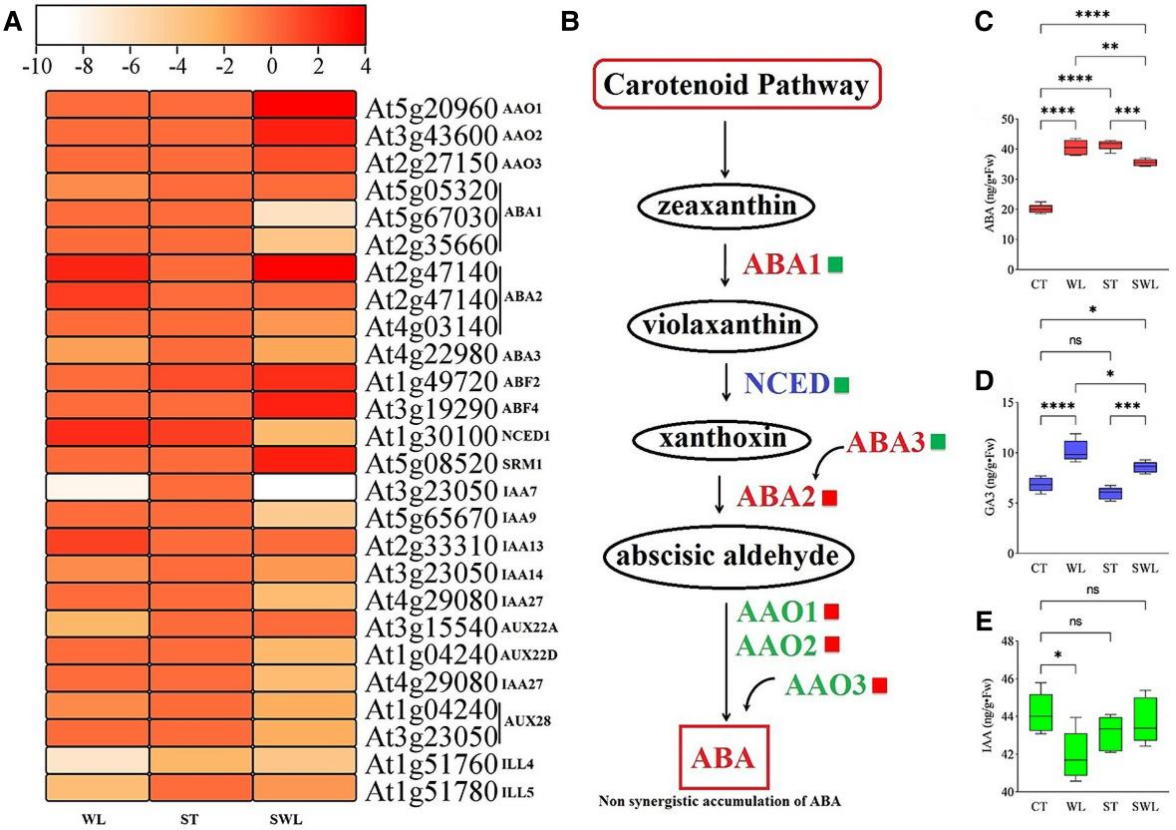
Plant hormones variations in fragrant rosewood under single and combined stresses. A Heat map of the ABA biosynthesis genes **A)**, schematic diagram representing the nonsynergistic accumulation pathway of ABA under combined stress caused by an antagonistic regulation mode in crucial ABA-related genes; red squares represent a synergistic upregulation and green squares a synergistic downregulation **B)**. Variations in ABA **C)**, GA_3_**D)**, and IAA **E)** contents in fragrant rosewood leaflets subjected to single and combined stresses. Treatments are presented as follows: control (CT), salinity (ST), waterlogging (WL), and combination of WL and ST (SWL). The signs such as *, **, ***, ****, and NS represented the significance difference among different treatments. *P < 0.05, **P < 0.01, ***P = 0.0001, ****P < 0.0001; NS, not significant. Data are expressed as means ± Sd (5 replicates from 5 different seedlings), and significant differences between means were determined at a *P*-value of ≤0.05 with 1-way ANOVA test. The perpendicular line within each box represents the median value and the ends of the box represent the 3rd and 1st quartile. The median is the value separating the higher half from the lower half of data sample. The lower edge of the box is Q1, which is the median of the lower half of the dataset. The upper edge is Q3, which is the median of the upper half of the dataset.

**Figure 6.**
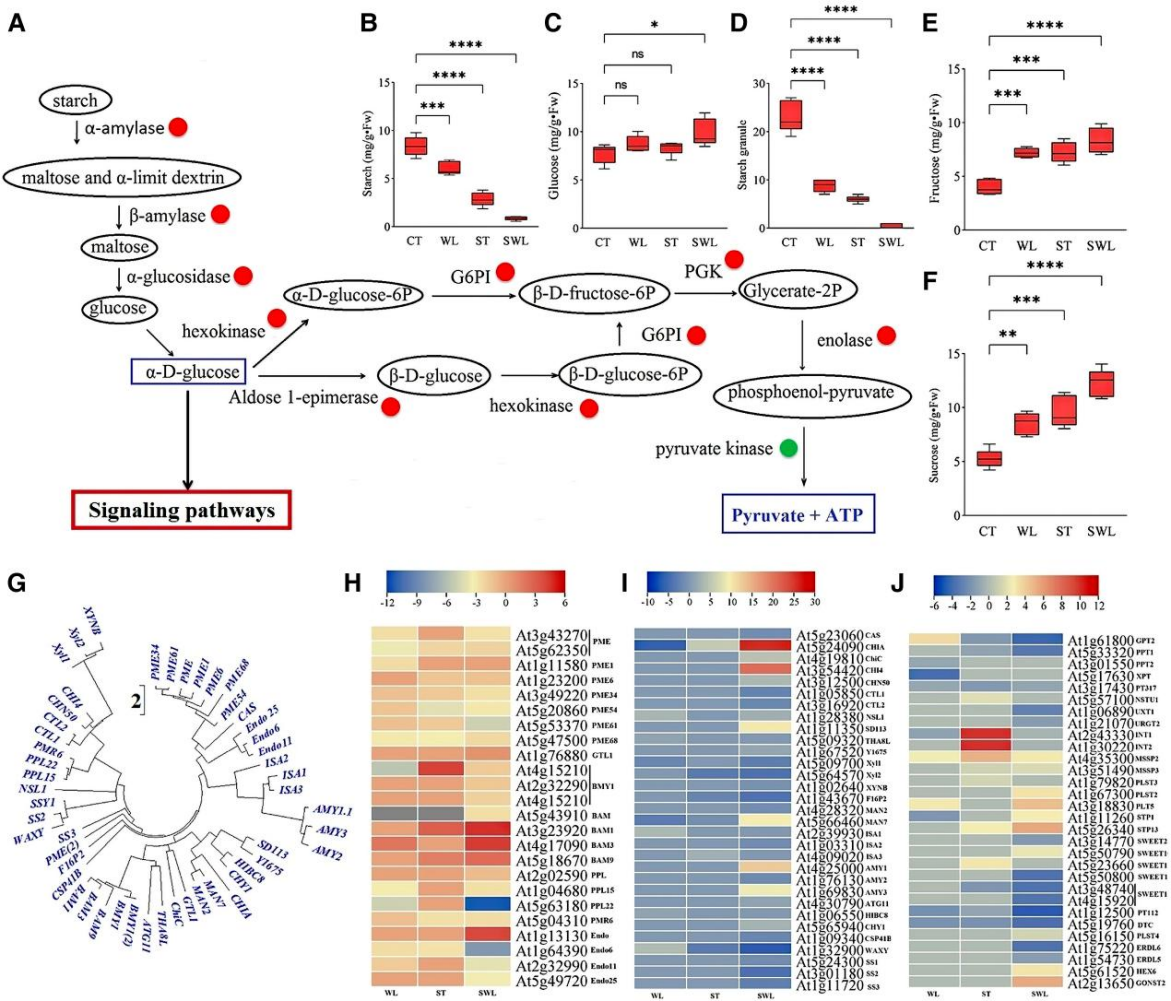
Carbohydrate degradation and biosynthesis in fragrant rosewood under single and combined stresses. A comprehensive diagram showing the mechanistic pathway of starch degradation and glycolysis in fragrant rosewood under combined stress, red circles represent a synergistic upregulation and green circles a synergistic downregulation **A)**. Variations in starch **B)** and glucose **C)** contents, number of starch granules (counted visually in TEM pictures) **D)**, fructose **E)** and sucrose **F)** contents. Carbohydrate biosynthetic pathway proteins-phylogeny tree based on the test Neighbour-Joining Tree and scale bar indicates number of changes per site **F)** and the heat maps of related genes in polysaccharide catabolism (**H** and **I**) and carbohydrate transport **J)**. Treatments are presented as follows: control (CT), salinity (ST), waterlogging (WL), and combination of WL and ST (SWL). The signs such as *, **, ***, ****, and NS represented the significance difference among different treatments. *P < 0.05, **P < 0.01, ***P = 0.0001, ****P < 0.0001; NS, not significant. Data are expressed as means ± Sd (5 replicates from 5 different seedlings), and significant differences between means were determined at a *P*-value of ≤0.05 with 1-way ANOVA test. The perpendicular line within each box represents the median value and the ends of the box represent the 3rd and 1st quartile. The median is the value separating the higher half from the lower half of data sample. The lower edge of the box is Q1, which is the median of the lower half of the dataset. The upper edge is Q3, which is the median of the upper half of the dataset.

SWL induced the synergistic pattern of the number of transcripts involved in ABA biosynthesis (Fig. 5, A and B) but a nonsynergistic pattern of ABA accumulation (Fig. 5C). Even though the basic leucine zipper class transcription factors (*ABF*) transcripts were upregulated in fragrant rosewood leaflets under SWL relative to under ST or WL (Fig. 5A) along with *ABA2* (xanthoxin dehydrogenase); abscisic-aldehyde oxidase-related genes (*AAO1*, *AAO2*, and *AAO3*); and crucial genes involved in ABA biosynthesis, such as *ABA1* (zeaxanthin epoxidase) and *ABA3* (molybdenum cofactor sulfur transferase) genes, the 9-*cis*-epoxy carotenoid dioxygenase (*NCED*) gene was synergistically repressed under combined stresses and induced under ST or WL. The gibberellic acid (GA_3_) content followed the same pattern as the ABA content under combined stress (Fig. 5D). Plant hormones, such as the auxin indole acetic acid content (IAA), and related genes showed also a nonsynergistic pattern under combined stress (Fig. 5, A and E). The perfect (positive association between number and regulation status) synergistic degradation of the polymeric carbohydrates (starch) in SWL-treated leaflets was correlated with an increase in monomers, such as glucose, sucrose, or fructose compared to control group (Fig. 6, A to F). However, glucose accumulation under WL or ST did not significantly decrease compared with that under SWL (Fig. 6C). Fructose and sucrose showed a synergistic increase under SWL in contrast to under ST/WL (Fig. 6, E and F). Figure 6G shows the phylogeny based on the respective protein sequences of several selected transcripts regulated under combined stress. Moreover, the main factors that trigger the repression of starch accumulation in SWL were related to the downregulation of genes related to *WAXY* (granule-bound starch synthase or *SS1-3*) and *glgA* (starch synthase) (Fig. 6, H to J). SWL repressed *SS1*, *SS2*, and *SS3*, which are involved in the synthesis of short glycan chains within amylopectin. Starch-debranching enzyme-related genes (*ISA1*, *ISA2*, and *ISA3*) involved in amylopectin biosynthesis were less affected by SWL than by other stresses. The specific effect of SWL was the suppression of genes encoding pectinesterases (pectin methylesterases; *PME*, *PME1*, *PME6*, *PME34*, *PME54*, *PME61*, and *PME68*) and endoglucanases (*Endo*, *Endo6*, *Endo11*, and *Endo25*), which function in the modification of cell walls via the dimethyl-esterification of cell wall pectin. However, several genes and their isoforms related to chitinase and β-amylase were strongly induced by SWL. The chloroplastic β-amylase genes (*BAM*, *BAM1*, *BAM3*, and *BAM9*) involved in the hydrolysis of alpha-D-glucosidic linkages in polysaccharides are a crucial step for monosaccharide formation and were significantly induced by SWL. The endohydrolysis of alpha-D-glucosidic linkages in the polysaccharide chain required a synergistic increase in 2 chloroplastic alpha-amylases (*AMY1* and *AMY3*). Monomer-facilitated transporters, such as *SLC2A13* (MFS transporter, SP family, solute carrier family 2 [myoinositol transporter], member 13), were found to play a more important role under SWL than under ST, WL, or CT. The significant repression of bidirectional sugar transporter genes (*SWEET 2*,*10*,*12*,*13*, and *17*) by SWL suggested weak interference in the low-affinity uptake and efflux of sugar across the plasma membrane.

### Emphasis on the oxidative transcriptional responses and physiological pattern under combined stress

**Figure 7.**
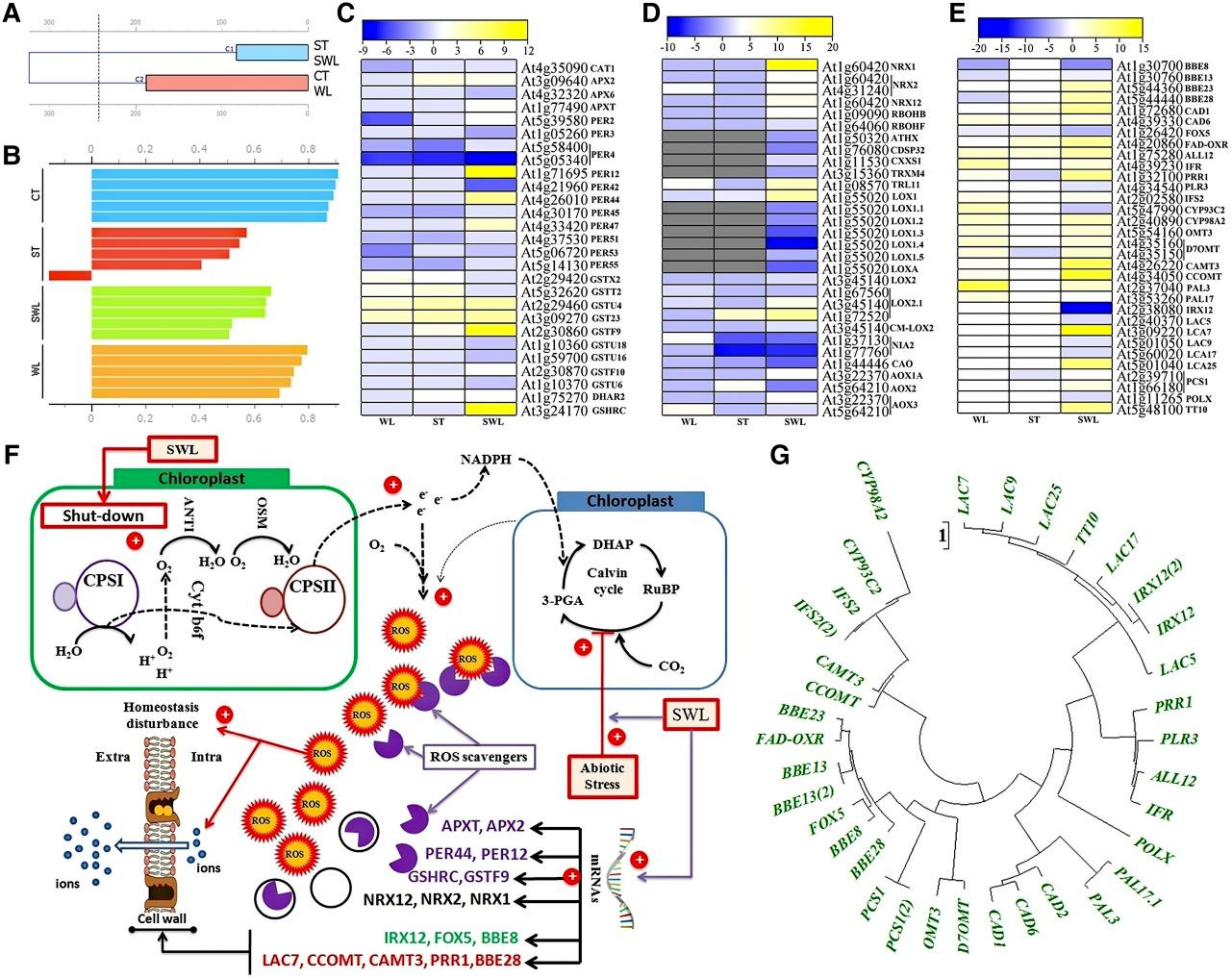
Biochemical and transcriptional profiles of oxidative stress in fragrant rosewood under single and combined stresses. Dendrogram of hierarchical clustering of the dataset regrouping different oxidative related-indices (H_2_O_2_, O_2_^•−^, POD, SOD, CAT and GPX, ASA and GSH, proline, phenols, flavonoids, PAL, and PPO) among CT, ST, WL, and SWL **A)** and silhouette analysis of the different clusters among different treatments based on Euclidean distance metric **B)**. Heat maps of genes in antioxidant activity, detoxification, and oxidoreductase activity **C)** and **D)** (the gray color in panel **D** represented the transcripts that were regulated only under combined stress), and phenylpropanoid pathway **E)** GO modules. Hypothetical schematic diagram showing the transcriptional regulation of oxidative stress under combined stress and the interactions with photosynthesis, Calvin cycle, cell wall biosynthesis, and homeostasis; red circles with a positive sign indicate a massive synergistic effect **F)**; ROS, reactive oxygen species; ANTI, antioxidants; OSM, osmoprotectants. Phenylpropanoid biosynthetic pathway proteins-phylogeny tree based on the test Neighbour-Joining Tree and scale bar indicates number of changes per site **G)**.

Oxidative items clustered into 2 separate groups, namely, ST and SWL, then in WL and SWL (Fig. 7A). Silhouette analysis revealed a cluster in the ST group with a negative value and other clusters with lower values (Fig. 7B), indicating that the antioxidative response under ST was more similar to that under SWL than to that under WL. Although the synergistic effect of combined stresses was predominant for each functional subset antioxidative response genes (Supplemental Fig. S9), the transcriptional and biochemical antioxidative responses showed a very complex pattern. Several genes, such as genes related to peroxidases (*PER12*, *PER44*, and *PER47*), ascorbate peroxidase (*APX2* and *APXT*), glutathione *S*-transferase (*GSTF9*, *GSTF10*, *GSTU4*, and *DHAR2*), glutathione transferase (*GST23*), and cytosolic glutathione reductase (*GSHRC*), presented a major involvement in ROS scavenging under combined stresses (Fig. 7, C to E). The emphasis between the decrease in photosynthesis (Fig. 4G) and ROS accumulation (Supplemental Fig. S9A) appeared to induce the significant upregulation of several mitochondrial ubiquinol oxidases (*AOX1A* and *AOX3*) in SWL-treated seedlings. Additionally, diverse probable thiol-disulfide oxidoreductase nucleoredoxins (*NRX1*, *NRX2*, and *NRX12*) were important components in SWL-treated seedlings (Fig. 7D). The fragrant rosewood is a medicinal plant with high concentrations of phenolics that play a crucial role in oxidative stress. The primary genes regulated in SWL and their patterns under single stresses are illustrated in Fig. 7, E and F to highlight the role of flavonoids and other phenolic compounds under SWL. Overall, the extensive synergistic pattern shown by the transcriptional changes in the phenylpropanoid biosynthesis pathway or antioxidative responses does not reflect the same extensive synergistic pattern exhibited by phenol, enzyme, or antioxidant molecule accumulation. Figure 7G illustrates the protein-phylogenetic structure of the targeted transcripts associated with antioxidative processes in fragrant rosewood when exposed to SWL.

### Does the plant morpho-anatomical analysis validate the metabolomic and transcriptional pattern results?

**Figure 8.**
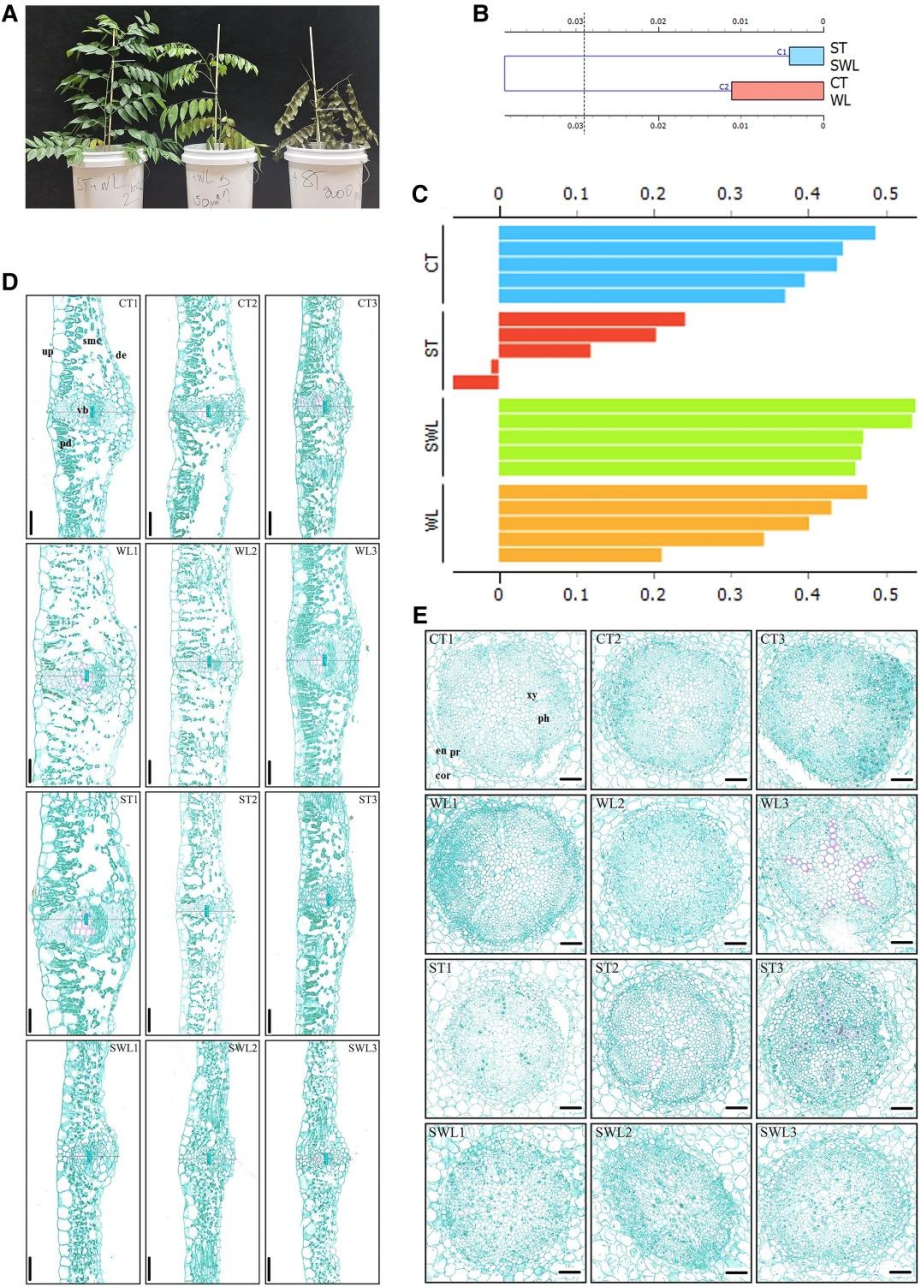
Plant morpho-anatomical responses of fragrant rosewood under single and combined stresses. Photograph of different levels of combined stress (SWL1, SWL2, and SWL3) effects on the shape of the seedlings **A)**. Dendrogram of hierarchical clustering of the dataset regrouping different morpho-physiological related-indices (plant shoot height, shoot and root biomass accumulation, root membrane permeability, shoot and root water contents, leaflet area, leaflet length, leaflet average width, leaflets max width, leaflet vascular bundle diameter and root stele diameter, etc.) among CT, ST, WL, and SWL **B)** and silhouette analysis of the different clusters among different treatments based on Euclidean distance metric **C)**. Anatomical changes of the leaflet (**D)** and root **E)** of fragrant rosewood exposed to CT, WL, ST, and SWL with 3 replicates each, row 1 (CT1, CT2, and CT3), row 2 (WL1, WL2, and WL3), row 3 (ST1, ST2, and ST3), and row 4 (WL1, WL2, and WL3). Leaflet images were taken at a magnification of 20× and 10× for roots, the zoom level was set with CaseViewer and scale bars = 50 *μ*m for leaflets and = 100 *μ*m for the roots. Abbreviations in panel **D** are: de, down-epidermis; pd, palisade mesophyll cell; smc, spongy mesophyll cell; ue, upper-epidermis; vb, vascular bundle. Abbreviations in panel **E** are: cor, cortex; en, endoderm; pr, pericycle; ph, phloem; xy, xylem. Treatments are presented as follows: control (CT), salinity (ST), waterlogging (WL), and combination of WL and ST (SWL).

Anatomical analysis was conducted on healthy leaflets. During the first experiment, the seedlings that were exposed to a combination of WL and 150 mM or 200 mM ST displayed severe damage after 1 wk of treatment as shown in Fig. 8A. Seedlings under WL combined with 100 mM ST were in good condition with healthy leaves. Hierarchical clustering revealed 2 groups comprising CT and a second group containing SWL, WL, and ST (Fig. 8B). Two clusters under the ST treatment exhibited a negative silhouette value that brought them closer to SWL than to WL (Fig. 8C). ST and WL presented different types of anatomical changes, including large (WL and ST, 1) and small surfaces (WL and ST, 2) of spongy and palisade mesophyll cells (Fig. 8D). The size and organization of the vascular bundle were more consistent in different samples from the CT group. At this stage, determining a pattern under SWL on the basis of the leaf anatomy observations under ST and WL is difficult. The stele that comprised all the tissues inside the endodermis (xylem, phloem, and pericycle) is shown in Fig. 8E to depict the effect of SWL on root anatomy relative to that of CT and single stresses. The stele is the pivotal part of the root system that contains vascular tissue (xylem and phloem). The seedlings under CT maintained larger (*P* < 0.05) stele areas (Supplemental Fig. S10) than those under the SWL treatment, which showed the same trend as the seedlings under ST and WL. However, the difference between the SWL and ST groups and that among ST, WL, and SWL were not statistically significant. Root and leaf morpho-physiology (Supplemental Figs. S11 and S12) were analyzed to further confirm the pattern of leaf and root anatomical variation under SWL. The results revealed a strong decrease in shoot height, leaf area, leaf vascular bundle diameter, and shoot water content under SWL (Supplemental Fig. S12, A and D). Root membrane permeability (Supplemental Fig. S12E) increased significantly (*P* < 0.05) under ST, WL, and SWL. The pattern under SWL was synergistic in contrast to that under single stresses. Rootwater content showed the same trend and similar variations between ST and WL (Supplemental Fig. S12F).

## Discussion

### Uniqueness of the molecular responses of plants under combined stress

The responses of the molecular profiles of plant species subjected to various combinations of environmental stresses differ from stress to stress (Zandalinas and Mittler 2022). The combination of ST, drought, and heat stresses and cold or high light resulted in a lower percentage of similarity in transcript expression than a single stress. Metabolomic changes showed the same pattern (Mittler 2006). We observed the same pattern in SWL-treated seedlings, which showed a more impressive increase in expressed transcripts than seedlings exposed to WL (241.8%) or ST (426.3%). The uniqueness of the metabolomic response of fragrant rosewood (*D. odorifera*) leaflets under SWL (Supplemental Tables S4 and S5) was based on the repression of lipids, such as glycolipids comprising monogalactosyldiacylglycerol (18:20, isomer2) and digalactosyl–diacylglycerol (18:1). The upregulation of these glycolipids under WL or ST would be related to their stress-related functions in membrane stabilization and stress signaling (Zhang et al. 2019). The singularity of the metabolomic responses of plants under SWL was also based on the expression of phenylalanine, tyrosine, aspartic acid, pyrrole-2-carboxylic acid, histidine, aminoadipic acid, aspartic acid, and methionine, which were upregulated by SWL. At the same time, they were suppressed under WL or unchanged under ST. The accumulation of amino acids in plants under harsh conditions is a requirement for an alternative respiratory substrate (Batista-Silva et al. 2019). We observed the same phenomenon in transcriptional response, whereby the expression of numerous distinct genes was exclusively induced in seedlings under SWL, leading to unique physiological changes. Notably, the upregulation of specific genetic loci (At4g19170, At3g63140, At1g09340, At3g27925, or At2g47940) involved in plastoglobulus formation demonstrated a strong association with the accumulation of OP in chloroplasts as confirmed by transmission electron microscope (TEM) analysis. The combined effect of stressors produced a unique metabolomic and transcriptomic signature, potentially leading to uncommon physiological alterations. The results provided by TEM analysis revealed a synergistic accumulation of chloroplastic OP that was associated with a synergistic decrease in root and leaf anatomy (Fig. 8) and morphology (Supplemental Figs. S11 and S12), which is a basic characteristic of combined stress. The unique transcriptional and metabolomic responses of plants under combined stresses result in drastic changes in plant morphology and anatomy.

### Combined stresses induced a mosaic of pattern modes of commonly expressed transcripts and metabolites

The transcripts or metabolites with a pivotal role in plant breeding and genetic research on multifactorial stress tolerance are those expressed under ST, WL, and SWL. Elucidating their expression patterns under combined stresses is critical to identify gene markers or candidates that exhibit high efficacy under single and combined stress conditions. The visualization of common possible molecular marker patterns allows us to predict the behaviors of multiple metabolites and/or genes accurately in plants exposed to combined stresses. The results of a previous study revealed that transcripts that react solely to combined stresses, rather than to individual stress factors, exhibit unpredictable behavior (Rasmussen et al. 2013). Our present study demonstrated that the neutral mode was the most prevalent mode of common metabolites and transcripts. These markers can have a substantial effect either individually or under combined stress conditions. While their high expression under WL, ST, or SWL may confer considerable tolerance, several of these markers exhibited low expression levels under all treatments, indicating a reduced role in stress-responsive systems activated by single or combined stresses. Multifactorial stress is expected to increase plant response at the metabolomic and transcriptional levels (Mittler 2006), and this phenomenon should be reflected by the synergistic pattern mode. The massive increase in stress-responsive genes was the perfect example of the synergistic pattern imposed by combined stress in fragrant rosewood leaflets (Supplemental Table S6). However, the common transcriptional response of fragrant rosewood leaflets showed a more remarkable synergistic pattern than the metabolome. These gene behaviors are due to the intensity of stress in plants, suggesting that these behaviors can be predicted in plants under combined stresses. Transcripts of acyl-CoA oxidase (*ACOX1* and *ACOX3*), omega-6 fatty acid desaturase (*FAD2*), or asparagine synthase (*ASNS*), which are crucial in plant stress tolerance, presented a synergistic pattern under SWL. Although we found common metabolites with an antagonistic pattern, the transcriptional pattern showed a quasi-null number of transcripts in the antagonistic pattern mode. The enhancement in the antagonistic mode in plants under combined stresses can arise from diverse signaling pathways that interact and restrain each other, a mechanism that may be intensified under a combination of stresses (Suzuki et al. 2014; Shaar-Moshe et al. 2017). The common transcripts or metabolites presenting a dominant, minor, or unilateral mode maybe induced either by ST or WL in response to combined stresses.

### Shutdown of the photosynthetic apparatus under combined stresses and the less-needed role of abscisic acid (ABA) in the signaling pathway under SWL

PSII, as a multi-subunit enzymatic complex that catalyzes the breakdown of water molecules during photosynthesis (Rhee et al. 1998), showed the same pattern as PSI. The high repression frequency of *LHCBs* in PSII was associated with a significant decrease in ABA content in SWL compared to ST or WL. The over-expression of the *LHCB6* gene in *Arabidopsis* induced ABA accumulation, which favored the stomatal response (Xu et al. 2012). The ability of the ABA receptor to trigger downstream signaling cascades to induce photosynthetic changes is strongly dependent on the regulation of *LHCBs* (Xu et al. 2012). A previous report mentioned that genes related to *ABA1*, *ABA3*, and *AAO3* might be highly sensitive to ABA-mediated positive feedback in ABA biosynthesis (Xiong et al. 2002). The decrease in *ABA1* and *NCDE* would explain a considerable part of the mechanistic molecular pathway behind the decrease in ABA content under SWL relative to that under ST or WL despite their synergistic pattern. The results highlighted that *ABA1* might have a more important function than*ABA2* or *ABA3* in ABA regulation under SWL. *ABA1* genes have been well established to catalyze the epoxidation of zeaxanthin into antheraxanthin and all-*trans*-violaxanthin, which is converted into the *cis*-isomers of neoxanthin and violaxanthin cleaved by *NCED* to form xanthoxin. Xanthoxin is converted into abscisic aldehyde, which is an intermediate product in the biosynthesis of ABA (Schwartz et al. 1997; Qin and Zeevaart 1999; Perreau et al. 2020). Moreover, our results suggested that under combined stresses, the synergistic accumulation of ABA in plant leaflets is not required to regulate stomata and net photosynthetic rates. In addition, the upregulation of several *PSAs* and *PSBs* in *Arabidopsis* under heat combined with high light (Balfagón et al. 2019) contradicted the results found in fragrant rosewood under SWL. ABA is well known to play a crucial role in the plant signaling response against osmotic stress; such response is mainly triggered in plants by abiotic stresses through the regulation of a broad spectrum of osmotic stress-responsive genes (Sies et al. 2017). The upregulation of ROS was more important than that of ABA under SWL. ABA concentration under SWL was lower than that under ST or WL. This finding suggests that ABA has a small role in the signaling response of fragrant rosewood seedlings under SWL. The plant mitogen-activated protein kinase (MAPK) signaling pathway map revealed (Supplemental Fig. S13) that SWL induced more genes involved in hydrogen peroxide (H_2_O_2_) or ethylene signaling pathways in stress tolerance responses or adaptation than in ABA. Several plant *MAPKs* are triggered by various abiotic stresses (Zhang and Klessig 2001), and our results suggest the important function of *MAPKs* and ROS in the SWL signaling response. Although ABA content and ABA-related genes were associated with reduced ABA content in fragrant rosewood leaflets under SWL, plant hormone signal transduction-related genes were significantly enriched (Supplemental Fig. S14). Given that IAA and gibberellin (GA_3_) contents did not massively increase under SWL, other plant hormones, such as ethylene as suggested by the MAPK map, would play a crucial role in SWL tolerance.

### Starch degradation and plastoglobulus synthesis emerged to play a crucial role in fragrant rosewood chloroplasts under SWL

The shutdown of the photosynthesis apparatus by SWL was also applicable to *ABC1* genes (*ADCK*; aarF domain-containing kinase). In transgenic plants, the loss of function of the *ABC1* gene strongly weakened photosynthetic performance and stress tolerance (Espinoza-Corral and Lundquist 2022). Several ABC1 proteins have been identified in *Arabidopsis* chloroplasts, specifically in the plastoglobulus proteome (Lundquist et al. 2012, Martinis et al. 2013), suggesting that ABC1-like kinase has a stabilizing function in *Arabidopsis* plastoglobuli. Under SWL, the repression of *ABC1* genes was associated with a remarkable increase in OP in fragrant rosewood chloroplasts, confirming the stabilizing function of ABC1 proteins in plastoglobulus formation. Moreover, stress-induced starch degradation is a well-known pathway in plant stress tolerance acquisition and improvement (Thalmann and Santelia 2017). β-Amylase, which was massively upregulated under SWL, is a main enzyme in starch degradation. In transgenic potato leaves, β-amylase deficiency indicated a defect in starch degradation (Scheidig et al. 2002). WL combined with different ST concentrations has been shown to improve sugar monomer accumulation in Herbaceous seepweed (*Suaeda maritima*) compared with single-stress conditions (Behr et al. 2017). The same pattern was found in maize under combined cold and drought stresses (Guo et al. 2021) and in *Arabidopsis* under drought and heat (Prasch and Sonnewald 2013). Furthermore, the role of sugar transporters under combined stresses is largely unexplored. The present study acknowledges a massive synergistic pattern in transcripts involved in carbon fixation, carbon metabolism, and starch and sucrose metabolism in fragrant rosewood under combined stress (Supplemental Fig. S15).

### Nucleoredoxin-related transcripts may maintain a high synergistic antioxidative system in fragrant rosewood leaflets under combined stress

Oxidative stress is rarely discussed in the small number of scientific studies that investigated the transcriptional responses of plants to combined stresses. The overproduction of H_2_O_2_ and superoxide anions (O_2_^•−^) in fragrant rosewood under ST, WL, or SWL is related to the various functions of ROS in stress tolerance, such as signaling pathways or programmed cell death. ROS-induced DNA, lipid, or protein damage is related to defective detoxification (scavenging or quenching) (Foyer and Hanke 2022). The strong upregulation of ROS-response transcripts in *Arabidopsis* under the combination of high light and heat stresses described by Zandalinas et al. (2020) showed an important key role of ROS in plant systemic signals and responses under combined stress. However, these results highlighted only the function of ROS in the plant signaling response under stress integrated with salicylic acid-response transcripts. We found the dual regulation of peroxidase and L-ascorbate peroxidase-related transcripts (up- and downregulation) under combined stresses and the upregulation of most glutathione *S*-transferase-related transcripts. The impressive number of upregulated nucleoredoxin-related transcripts was a main factor that led to antioxidant system defense against oxidative stress in fragrant rosewood leaflets under SWL (Supplemental Fig. S16). In *Arabidopsis* plants, Kneeshaw et al. (2017) exposed a determinant function of *NRX1*, a member of the TRX superfamily of enzymes in maintaining catalase enzymes in a reduced state, thereby protecting their H_2_O_2_-detoxifying activity, which safeguarded the antioxidant system during oxidative stress. Crosstalk between oxidative stress and phenylpropanoid pathways under combined stresses is scarce. The contents of phenols, flavonoids, and enzymes involved in the phenylpropanoid pathway (phenylalanine ammonia-lyase and polyphenol oxidase) increased under ST or LW and SWL (Supplemental Fig. S17). However, we did not find a synergistic pattern in the biochemical and metabolomic characterizations of phenylpropanoid pathways. Fragrant rosewood leaflets showed synergistic transcriptional responses under SWL but not under single stresses. The transcriptional response was not reflected by the biochemical and metabolic characteristics under SWL mainly because several gene keys and their isoforms involved in the phenylpropanoid pathway, such as laccase-related genes, were downregulated.

## Conclusions

Plants facing combined stresses follow a general principle that is based on a synergistic transcriptional response due to 2 factors: (i) the intensity of stress experienced by the plants that is related to the simple addition of 1 stress to another (ii) and the integration of each specific response related to the specific tolerance mechanism for each single stress participating in the combination of stresses. In this study, we experimentally deciphered the different adaptive molecular mechanisms involved in the SWL responses of fragrant rosewood seedlings after non-acclimation stress. Compared with a single stress, combined stresses triggered an impressive number of transcripts and DMs. The main biological functions discussed in the present study revealed the synergistic responses of fragrant rosewood leaflets at some points under SWL. The analysis of individual transcript or metabolite patterns revealed 7 different modes, among which the neutral mode was the most represented. Our analysis of the biological function of metabolites and transcripts showed that the synergistic effect of SWL on the biological function or type of parameters studied is not a general rule in plants. ABA, which is a crucial plant hormone related to stress tolerance and signaling pathways, decreased in SWL-treated seedlings relative to in ST- or WL-treated seedlings. The *ABA*-related transcripts *ABA1* and *NCDE* showed the same trend as ABA and were downregulated. Nevertheless, ABA was significantly higher under SWL than under CT. This situation partly permitted a low variation in net photosynthetic and stomatal conductance in SWL-treated seedlings. The SWL-triggered repression of *LHCBs* and photosystem subunit-related genes and isoforms in PSI and PSII appeared to be the main factor of the shutdown of the photosynthetic apparatus. The dynamic coordination in the response of fragrant rosewood leaflets to SWL was characterized by the extensive degradation of starch into sugar monomers through β-amylase along with the increase in sugar transporters, such as *SLC2A13*. The same trend was also observed in the analysis of antioxidative system defense against oxidative stress. Collectively, shoot biomass accumulation, water content, and height; leaf area; root anatomy; membrane permeability; and water content verified the transcriptional variation in fragrant rosewood under SWL. Knowledge is changing and climate change is exacerbating rapidly. Therefore, the scientific community is urged to provide a meaningful molecular database to enhance every new concept of plant stress tolerance, thus making each contribution a bit like a grand experiment.

## Materials and methods

### Plant material and experimental setup

The fragrant rosewood (*D. odorifera*) is a species endemic to Hainan with considerable medicinal value. Two-year-old saplings of fragrant rosewood (500 seedlings) were purchased from a wholesale plant nursery located in Ledong County (18°42′57.91″N, 108°52′18.65″E), Hainan Province, China. Seedlings grown in a plastic bag were moved into plastic pots filled with red soil, sand, and coconut coir (2:2:1, v/v/v). The experiment was conducted from March to July. Physicochemical analysis showed that the red soil contained 5.34 mg kg^−1^ammonia nitrogen, 11.78 mg kg^−1^ available phosphorus, and 81.72 mg kg^−1^ available potassium. The seedlings were cut off at 10 cm from the root. The pots were transferred to a shaded environment in a greenhouse located at Hainan University (20°03′22.80″N, 110°19′10.20″E). After young leaves approximately 5 cm in length appeared, the seedlings were placed under normal light conditions and watered every day for 2 mo. A total of 320 healthy seedlings with approximately the same size were divided into 2 experimental groups. The first experiment was set up with 200 samples in a completely randomized trial in which different stressors were tested for 14 d. The trial comprised 8 treatments (25 seedlings per treatment), including a control treatment (well-watered conditions; CT). The salt treatment was tested at 3 levels: ST1 (100 mM NaCl), ST2 (150 mM NaCl), and ST3 (200 mM NaCl). ST treatments were first imposed at 100% field capacity. Then, ST-treated seedlings were watered with 100 mL of saline solution every 2 d during the rest of the trial. For the WL treatment, the pots containing seedlings were partially submerged in aqueous solution in a 10 L plastic bucket. Then, the water level was maintained at 10 cm above the soil surface. The SWL treatment included SWL1 (ST1 + WL), SWL2 (ST2 + WL), and SWL3 (ST3 + WL). The pots were placed in the same plastic bucket as WL, and 100 mM NaCl solution was added to replace the evaporated solution. After 7 d of the experiment, the whole SWL solution was replaced with a new solution for the first experiment. The stress damage index (SDI) was used to select the appropriate experimental design on the basis of the phenotypic responses of the seedlings exposed to different treatments. The method was applied in accordance with Falakboland et al. (2017). The SDI was based on visual observations related to chlorosis and necrosis in fragrant rosewood leaflets under CT, ST1-3, WL, and SWL1-3. The scoring system was between 0 and 10; leaflets presenting no visual symptoms were given a score of 0 and dead seedlings were given a score of 10 (Supplemental Table S7). The water potential of 3 leaves (at the top) sampled from each treatment was measured 1 h after the stressors were applied for 7 d by using a Dew-point PotentiaMeter WP4 (Gene Company Ltd, USA). The values are reported in Supplemental Table S8. Survival rate was determined after 2 wk of treatment and is shown in Supplemental Table S8. During the first experiment, the responses of fragrant rosewood to SWL2 and SWL3 at early stages were not an adaptive mode but a survival mode. The only adaptive response was found in SWL1. From Day 3 as shown in Supplemental Figs. S18 to S20, there was a massive amount of unhealthy leaflets with chlorosis and necrosis in SWL2 and SWL3. Moreover, the level of proline in SWL2 and SWL3 was lower than the CT at Day 6 (Supplemental Fig. S20) which showed a severe decline of the adaptive stress mechanism of fragrant rosewood seedlings to SWL. Thus we could not use WL + 150 mM or WL + 200 mM of NaCl in the present study. Indeed, the purpose of the present study is to compare 3 different stress factors, one of which was the combination of 2 single stresses based on an adaptive response. And, one of the innovations in the present manuscript is the choice of the treatment in the second experiment based on the results from the first experiment. Indeed, it has been well established that salinity at different concentrations (100 mM, 150 mM, or 200 mM) will induce gradually several adaptive salt-responsive genes. To make sure that we triggered a large number of salt-responsive metabolites and transcripts, we have chosen 200 mM over 100 mM at Day 6 in the second experiment. Indeed, at Day 6, 100 mM of salinity showed biochemical results (proline), dew-point water potential (DWP), or plant survival rate close to those in control as showed in Supplemental Table S8. Experiment 2 was set up on the basis of the results of the first experiment. The sampling time point was chosen before the first appearance of visual stress symptoms and at a high DWP to observe the responses caused specifically by the combination of WL and ST conditions. The SDI shown in Supplemental Fig. S18 and the visual morphology of fragrant rosewood leaflets is presented in Supplemental Fig. S19 and the proline variations showed in Supplemental Fig. S20 were used as the basis of the selection of the most suitable combination mode of SWL. Four treatments (30 replicates each), including CT, WL, ST3, and SWL1, were performed during the second experiment, and healthy fresh samples were harvested on Day 6. Further physiological and molecular data analyses related to the second experiment are provided in Supplemental Figs. S21 to S29.

### Morphological and physiological characterizations

Morphological traits were measured on Day 6. Biomass accumulation (roots and leaves), leaflet surface indices, and water status were determined with 5 representative replicates (*n* = 5). Leaflet area, length, and width were measured from mature leaflets at the top of the plant with a portable area meter LI-3000C (Li-COR, Lincoln, USA). Leaflet water content (LWC) was determined by the following formula: LWC (%) = (FW − DW)/FW × 100. Fresh leaflets were weighed (FW) then dried at 80 °C for 48 h. The dried material was weighed, and its weight was recorded as DW. Cell death in roots was analyzed spectrophotometrically with the method described by Jacyn Baker and Mock (1994) with Evans blue solution.

Photosynthetic parameters were measured from 9 AM to 12 PM with a LI-COR 6400 portable photosynthesis system. The levels of phytohormones, such as abscisic acid (ABA), auxin (IAA), and gibberellin (GA_3_), were determined as described in previous research (Cisse et al. 2022). Nonstructural carbohydrates (sucrose, fructose, and glucose), starch, and proline were measured as described by Pu et al. (2021). The levels of oxidative stress-related enzymes (peroxidases, superoxide dismutase, phenylalanine ammonia-lyase, polyphenol oxidase, and glutathione peroxidase), molecules (flavonoids, phenols, ascorbic acid, reduced glutathione, and alternative oxidase proteins), and hydrogen peroxide (H_2_O_2_) were determined by applying Solarbiocolorimetric assay kits (Supplemental Table S9). The details related to each assay kit are provided in Supplemental Table S3. Superoxide anions (O_2_^•−^) were measured as described by Cisse et al. (2022).

### Safranin fast green and transmission electron microscope staining

Leaflets, including major veins and roots (*n* = 6) collected on Day 6 were cut and fixed in 50% (v/v) FAA fixative solution for 24 h. The samples were deparaffinized with pure BioDewax and Clear Solution for 20 min, with pure ethanol for 5 min, and finally with 75% ethanol. The slides were stained with fast green dye solution for 1 to 5 min, washed, and soaked in 1% hydrochloric acid and alcohol for 10 s. Thereafter, the slides were stained with Safranin dye solution for 1 to 5 s then placed in100% ethanol for dehydration. Finally, the slides were immersed in xylene for 5 min and sealed with neutral resin. The samples were observed under an orthostatic microscope (Nikon Eclipse E100, Nikon, Japan), and images were analyzed with CaseViewer software (3DHISTECH Ltd., Budapest, Hungary).

Fresh leaf tissues of fragrant rosewood under different stress treatments were analyzed by using a TEM to visualize the variations in chloroplasts, starch granules, and plastoglobuli. Briefly, a sharp blade was used to harvest 1 mm^3^ of a sample, which was transferred into an EP tube with fresh TEM fixative (Servicebio, G1102) for fixation. The samplings were placed at room temperature for 2 h and fixed at 4 °C. Thereafter, the tissues were washed by using 0.1 M phosphate buffer (PB) (pH 7.4) 3 times (15 min each). Approximately 1% osmium tetroxide (OsO_4_) in 0.1 M PB (pH 7.4) was used for post-fixation for 7 h at room temperature. Then, OsO_4_ was removed with 0.1 M PB (pH 7.4) 3 times (15 min each). The samples fixed in OsO_4_ solution were dehydrated in graded ethanol and an ethanol:acetone mixture successively. The ethanol series comprised 30%, 50%, 70%, 80%, 95%, 100%, and 100% ethanol. The samples were soaked in each ethanol concentration for 1 h. Then, ethanol:acetone (3:1, 0.5 h), ethanol:acetone (1:1, 0.5 h), ethanol:acetone (1:3, 0.5 h), and pure acetone were added for 1 h. Resin penetration and embedding were performed with pure EMBed 812 (SPI, 90529-77-4) and a series of mixed solutions (acetone and EMBed 812) as follows: acetone:EMbed812 (3:1) for 2 to 4 h at 37 °C, acetone:EMbed812 (1:1) overnight at 37 °C, acetone:EMbed812 (1:3) for 2 to 4 h at 37 °C, and 100%EMBed 812 for 5 to 8 h at 37 °C. Embedded samples were transferred into a 65 °C oven (48 h) for polymerization. Subsequently, blocks were cut into 60 to 80 nm thin sections with an ultramicrotome (Leica UC7). Tissues were transferred onto 150 mesh cup rum grids with formvar film. Alcohol solution saturated with 2% of uranium acetate (avoiding light staining) was used for staining for 8 min. Then, the cup rum grids were rinsed with 70% ethanol 3 times and with ultrapure water 3 times. Subsequently, 2.6% lead citrate (avoiding CO_2_ staining) was performed for 8 min. Finally, the samples were rinsed with ultrapure water 3 times. The cup rum grids were dried, observed, and imaged with TEM (HITACHI, HT7800/HT7700).

### Widely targeted metabolomics assay

Freeze-dried leaf samples were crushed into powder by using a mixer mill (MM 400, Retsch) with zirconia beads for 1.5 min at 30 Hz. Approximately 100 mg of powder was weighed and extracted at 4 °C with 1.0 mL of 70% aqueous methanol then centrifuged at 10,000 × *g* for 10 min. The sample extracts were absorbed (CNWBOND Carbon-GCB SPE Cartridge, 250 mg, 3 mL; ANPEL, Shanghai, China) and filtered (SCAA-104, 0.22 *μ*m pore size; ANPEL) before LC‒MS analysis. An LC‒ESI‒MS/MS system was used to analyze the extracts, and the analytical conditions were as follows: HPLC column, Waters ACQUITY UPLC HSS T3 C18 (1.8 *μ*m, 2.1 mm × 100 mm); solvent system, water (0.04% acetic acid):acetonitrile (0.04% acetic acid); gradient program, 95:5 V/V at 0 min, 5:95 V/V at 11.0 min, 5:95 V/V at 12.0 min, 95:5 V/V at 12.1 min, 95:5 V/V at 15.0 min; flow rate, 0.40 mL/min; temperature, 40 °C; and injection volume: 2 *μ*L. The effluent was connected to an electrospray ionization (ESI)-triple quadrupole-linear ion trap (Q TRAP)-MS. Triple quadrupole (QQQ) scans and LIT were acquired on a Q TRAP mass spectrometer (API 6500 Q TRAP LC/MS/MS System). The system was equipped with an ESI Turbo Ion-Spray interface and operated in positive-ion mode and managed by Analyst 1.6.3 software (AB Sciex). The ESI parameters were set as follows: the collision gas (N_2_) was set at 5 psi; the ion spray voltage was 5500 V; the source temperature was 500 °C; and the ion source gas I, gas II, and curtain gas were set at 55, 60, and 25.0 psi, respectively. QQQ scans were performed as multiple reaction monitoring (MRM) experiments with nitrogen as the collision gas. The declustering potential (DP) and collision energy (CE) for individual MRM transitions were determined with further DP and CE optimization. For each period, a specific set of MRM transitions was monitored in accordance with the metabolites eluted within this period. The secondary spectral data obtained were qualitatively analyzed on the basis of public metabolite databases (MassBank, KNApSAcK, Metlin, MoTo DB, and hmdb) and a self-built MetWare database. Differential metabolite (DM) annotation and metabolite enrichment pathway analyses were performed with the Kyoto Encyclopedia of Genes and Genomes (KEGG) database (http://www.genome.ad.jp/kegg/).

### Transcriptomic analyses

RNA-seq was performed to dissect the global transcriptional adaptations in fragrant rosewood leaflets under single and combined stresses. The transcriptomic assay involved RNA extraction and sequencing and data processing and analysis. It was performed with 4 replicates of fresh mature leaves from the top of a plant. Briefly, fresh leaf samples were harvested on Day 6 from 4 replicates and immediately frozen in liquid nitrogen, and the total RNA was extracted by using aTRIzo Kit (Promega, Beijing, China). Sample contamination and RNA purity were assessed by using a NanoPhotometer (Thermo Fisher Scientific) at the optical densities of 260/280 and 260/230, respectively. RNA integrity was detected accurately with an Agilent 2100 Bioanalyzer (Agilent Technologies, CA, USA). Poly(A) mRNA was isolated from total RNA samples with magnetic oligo (dT) beads and used for mRNA-sequencing library construction. Library fragments were purified with an AMPure XP system (Beckman Coulter, Beverly, MA, USA) to select cDNA fragments (preferentially 250 to 300 bp in length). The usability of the RNA-seq data was verified through reverse transcription quantitative PCR. mRNA-sequencing libraries were sequenced by using an Illumina HiSeq 2500 sequencing platform (Illumina Inc., San Diego, USA). The data obtained from the sequencer were converted into sequence data (raw reads) by using CASAVA and stored in FastQfile format. Sequencing data were filtered by applying an ultrafast all-in-one FASTQ preprocessor (fastp) in accordance with (Chen et al. 2018) to ensure the reliability of bioinformatics analysis and obtain high-quality data for subsequent analysis.

The base quality of each sample was assessed by using FastQC (http://www.bioinformatics.babraham.ac.uk/projects/fastqc). Trinity was employed to assemble the filtered high-quality sequencing data to obtain the transcriptome, which was used as a reference sequence for subsequent differential expression analysis. Unigenes were extracted from the transcriptome, and the relevant information on the transcripts and unigenes was obtained as shown in Supplemental Table S1. Coding sequence prediction was performed before gene function annotation. Six databases, namely, KEGG, GO, NR, Swiss-Prot, trEMBL, and KOG, were used to annotate the predicted protein sequences. The conditions used for the BLAST comparison database were E-value below 1e^−5^, identity above 30%, and sequence coverage above 30%. The software package RNA-Seq by Expectation Maximization (RSEM) was set up to quantify gene and isoform abundances and the fragments per kilobase of transcripts per million fragments mapped (FPKM) of each corresponding gene. Differentially expressed genes (DEGs) were detected on the basis of the FC of the FPKM values. The false discovery rate (FDR) was fixed at ≤5% and used to define the *P*-value threshold (Love et al. 2014). The threshold for screening DEGs was arranged at an absolute value of log2FC and employed to define the *P*-value threshold (Benjamini and Hochberg 1995). The thresholding (clusterProfiler R package) and GO and KEGG pathway enrichment analyses of the DEGs were performed.

### Expression patterns, network visualization, and analysis

Hierarchical clustering was based on Euclidean distance. Then, Ward's linkage method was used to minimize the increase in the error sum of squares. A silhouette plot with a range between (−1, 1) was utilized to show the closeness of each group of parameters (*n* = 5) to its own cluster (group of data from 1 treatment) and to another cluster. If a coefficient is close to +1, then the cluster is distant from the other clusters belonging to the rest of the treatments. A null coefficient implies that the cluster is very close to another neighboring cluster, and a negative coefficient indicates that the data from 1 treatment belong to another cluster. This work provided a deep statistical and visual analysis of the global molecular pattern, including the metabolome and transcriptome, in fragrant rosewood under combined stresses. An interactive Venn diagram was used to identify common differential genes and metabolite expression. First, DMs were screened at FC ≥ 2 and ≤0.5. If the difference between the control and stress groups exceeded 2 in upregulated metabolites or less than 0.5 in downregulated metabolites, the difference between metabolites was considered significant. Subsequently, on the basis of the above description, selected metabolites with VIP ≥ 1 were chosen. The common transcript expression patterns were determined on the basis of log2FC and FDR in accordance with Shaar-Moshe et al. (2017). Each pattern found in the transcriptome and metabolome was classified as synergistic, additive, dominant, neutral, minor, unilateral, antagonistic, and non-assigned as shown in Fig. 1A. Red points represent upregulated metabolites or transcripts, and green points represent downregulated metabolites or transcripts. The standard deviation Sd (σ) was set at 0.50 on the basis of the Sd among the unique differential transcripts and metabolites in SWL (Supplemental Tables S10 and S11). Metabolites or transcripts assigned to synergistic, additive, and dominant modes were either up- or downregulated under single and combined stresses. The sum of the metabolite and transcript under a single stressor was calculated. The pattern mode under SWL was synergistic if the Sd between the sums was >0.50 and additive otherwise. On the basis of the Sd, if the metabolite or transcript log2FC under a single stress was equal to that under SWL, then the pattern is dominant. The neutral pattern mode corresponded to Sd ≤ 0.50 among the log2FC under each single stress relative to that under SWL whether they were either up- or downregulated. The minor combined-stress modes were determined on the basis of a lower (±Sd) log2FC than 1 or both single stresses. Metabolites and transcripts with opposite expression patterns were assigned to the unilateral or antagonistic mode. The mode is antagonistic if both expression patterns under a single stress are opposite those under SWL (±Sd). The unilateral mode included metabolites or transcripts under SWL with opposite expression patterns under a single stress (±Sd). Two-dimensional data projection with t-distributed stochastic neighbor embedding, Genes Network Explorer with Fruchterman–Reingold layout optimization, and correspondence analysis were performed with Python scripts integrated with Orange software (3.35.0). WGCNA was conducted in accordance with Langfelder and Horvath (2008) with R software and WGCNA package R scripts combined with Cytoscape as described by Shaar-Moshe et al. (2017). The protein phylogeny trees of different subsets of genes belonging to specific processes during SWL were generated with Molecular Evolutionary Genetics Analysis and through the test neighbor-joining tree function.

### Statistical analyses

Multivariate statistical analysis methods in Analyst 1.6.3. were used for mass spectral data processing, including PCA and orthogonal partial least squares discriminant analysis, in accordance with the method described by Eriksson et al. (2006). The data from metabolite analysis were normalized, and R software (www.r-project.org/) was used to perform hierarchical cluster analysis. The significant differences in data on physiological and morphological indices were found through 1-way ANOVA and Tukey's honestly significant difference test (GraphPad Prism 9.0.0). Data were expressed as the means ± SDs (5 replicates; 5 samples from 5 plants), and significant differences between means were determined at *P*-value of ≤0.05.

### Accession numbers

Raw sequencing files of mRNA sequencing are available at the National Center for Biotechnology Information, SRA database (https://www.ncbi.nlm.nih.gov/sra/) under accession number PRJNA1035943. The protein sequences used in the present manuscript file can be found in Supplemental Table S12.

## Supplementary Material

kiad639_Supplementary_Data

## Data Availability

All molecular (transcriptomic and metabolomic) data generated or analyzed during this study are included in this published article as supplementary excel files, the physiological and morphological data are available on request from the corresponding author.

## Supplemental data

The following materials are available in the online version of this article.

**Supplemental Figure S1.** Overview of the metabolomic data assessment in response to single and combined stresses.

**Supplemental Figure S2.** Summary of the transcriptomic data assessment in response to single and combined stresses.

**Supplemental Figure S3.** Volcano plots and MA plots of DEGs from fragrant rosewood under single and combined stresses.

**Supplemental Figure S4.** Weighted gene co-expression network analysis cluster dendrogram among waterlogging, salinity, and waterlogging combined with salinity in fragrant rosewood.

**Supplemental Figure S5.** Hub gene heat map of the weighted gene co-expression network analysis (Eigengene adjacency).

**Supplemental Figure S6.** Merged module tree of the weighted gene co-expression network analysis.

**Supplemental Figure S7.** Network heat map of the weighted gene co-expression network analysis and the different pattern mode found in fragrant rosewood under single and combined stresses.

**Supplemental Figure S8.** Donut-plots and heat map of different functional GO classifications related to DEGs belong to photosynthesis and carbon metabolism.

**Supplemental Figure S9.** Oxidative stress indicators and functional GO classification related-DEGs in antioxidant activity, detoxification, oxidoreductase activity, and phenylpropanoid pathway module.

**Supplemental Figure S10.** Average of root stele diameter variations in fragrant rosewood seedlings under CT, WL, ST, and SWL.

**Supplemental Figure S11.** Leaf morphology variations in fragrant rosewood leaflets under single and combined stresses.

**Supplemental Figure S12.** Leaf and/or root anatomy, morphology, and water status in fragrant rosewood seedlings under single and combined stresses.

**Supplemental Figure S13.** KEGG of plant MAPK signaling pathway in fragrant rosewood leaflet under combined stress.

**Supplemental Figure S14.** Heat map regrouping differentially genes enrichment following gene ontology biological function, the scale represents the number of transcripts and isoforms of each genes following GO classification.

**Supplemental Figure S15.** Heat map regrouping differentially genes enrichment following gene ontology biological function related to DNA, RNA activities, and other modules.

**Supplemental Figure S16.** Antioxidative enzymes and molecules variations in fragrant rosewood under single and combined stresses.

**Supplemental Figure S17.** Variations in proline, total of phenol, and flavonoids in fragrant rosewood under single and combined stresses.

**Supplemental Figure S18.** Variations of stress damage index in fragrant rosewood leaflets under single and combined stresses during the first experiment from Day 1 to Day 6.

**Supplemental Figure S19.** Pictures of fragrant rosewood at Day 6 under single and combined stresses.

**Supplemental Figure S20.** Variations in proline content in fragrant rosewood under single and combined stresses.

**Supplemental Figure S21.** GO classification for the differentially expressed genes in waterlogging-treated seedlings of fragrant rosewood.

**Supplemental Figure S22.** GO classification for the differentially expressed genes in salinity-treated seedlings of fragrant rosewood.

**Supplemental Figure S23.** GO classification for the differentially expressed genes in (salinity combined with waterlogging) SWL-treated seedlings of fragrant rosewood.

**Supplemental Figure S24.** GO enrichment scatter-plots of DEGs analysis between the control group versus salinity.

**Supplemental Figure S25.** GO enrichment scatter-plots of DEGs analysis between the control group versus waterlogging.

**Supplemental Figure S26.** GO enrichment scatter-plots of DEGs analysis between the control group versus salinity combined with waterlogging.

**Supplemental Figure S27.** Histogram of KOG classification.

**Supplemental Figure S28.** A pie chart of the statistical analysis of the number of different types of SSRs.

**Supplemental Figure S29.** Photographs of fragrant rosewood seedlings.

**Supplemental Table S1.** Global transcriptome details about fragrant rosewood leaflets.

**Supplemental Table S2.** Metabolomic responses pattern mode in fragrant rosewood leaflets under combined stress.

**Supplemental Table S3.** Common transcriptional (DEGs) response pattern modes in fragrant rosewood leaflets under combined stress.

**Supplemental Table S4.** Common metabolites downregulated under salinity (ST), waterlogging (WL), and salinity combined to waterlogging (SWL) in fragrant rosewood leaflets.

**Supplemental Table S5.** Common metabolites upregulated under salinity (ST), waterlogging (WL), and salinity combined to waterlogging (SWL) in fragrant rosewood leaflets.

**Supplemental Table S6.** Related transcripts and their isoforms classified in the module response to stress by GO under combined salinity and waterlogging (SWL).

**Supplemental Table S7.** Quantification of stress damage index in fragrant rosewood leaflets.

**Supplemental Table S8.** Physiological indices measured during the Experiment 1 (preliminary experiment) in fragrant rosewood seedlings under salinity (ST), waterlogging, and the combination of salinity and waterlogging (SWL).

**Supplemental Table S9.** Colorimetric assay kit details used during the experiment for biochemical characterization in fragrant rosewood leaflets under single (ST or WL) and combined stresses (SWL).

**Supplemental Table S10.** Transcriptional response pattern modes based on the selected modules (photosystem I, photosystem II, photosynthesis light harvesting, plastoglobule, polysaccharide catabolic process, and carbohydrate transport) related to the photosynthesis processes of GO enrichment classification.

**Supplemental Table S11.** Transcriptional response pattern modes based on the selected modules (antioxidant activity, detoxification, oxidoreductase activity, and phenylpropanoid biosynthetic process) related to the oxidative stress of GO enrichment classification.

**Supplemental Table S12.** All protein sequences and information used in the present manuscript.

## Dive Curated Terms

The following phenotypic, genotypic, and functional terms are of significance to the work described in this paper:

ABA1 Gramene: AT5G67030
ABA1 Araport: AT5G67030

ABA3 Gramene: AT1G16540
ABA3 Araport: AT1G16540

AAO3 Gramene: AT2G27150
AAO3 Araport: AT2G27150

ABA2 Gramene: AT1G52340
ABA2 Araport: AT1G52340

AAO1 Gramene: AT5G20960
AAO1 Araport: AT5G20960

AAO2 Gramene: AT3G43600
AAO2 Araport: AT3G43600

AMY1 Gramene: AT4G25000
AMY1 Araport: AT4G25000

AOX1A Gramene: AT3G22370
AOX1A Araport: AT3G22370

BAM3 Gramene: AT4G20270
BAM3 Araport: AT4G20270

GSTF10 Gramene: AT2G30870
GSTF10 Araport: AT2G30870

AMY3 Gramene: AT1G69830
AMY3 Araport: AT1G69830

APX2 Gramene: AT3G09640
APX2 Araport: AT3G09640

BAM1 Gramene: Solyc02g091840
BAM1 Araport: Solyc02g091840

DHAR2 Gramene: AT1G75270
DHAR2 Araport: AT1G75270

FAD2 Gramene: NCBI GeneID: 820387
FAD2 Araport: NCBI GeneID: 820387

pme1 Gramene: AT4G12390
pme1 Araport: AT4G12390

fructose CHEBI: CHEBI:28757
