# Supplementary material for: Dalbergia odorifera undergoes massive molecular shifts in response to waterlogging combined with salinity
Source: Plant Physiol. 2023 Dec 4;194(4):2301–21. doi: 10.1093/plphys/kiad639 (PMC10980518; doi:10.1093/plphys/kiad639)
Supplement: kiad639_Supplementary_Data [file kiad639_supplementary_data.zip › Revised Supplemetal Figures.pdf]

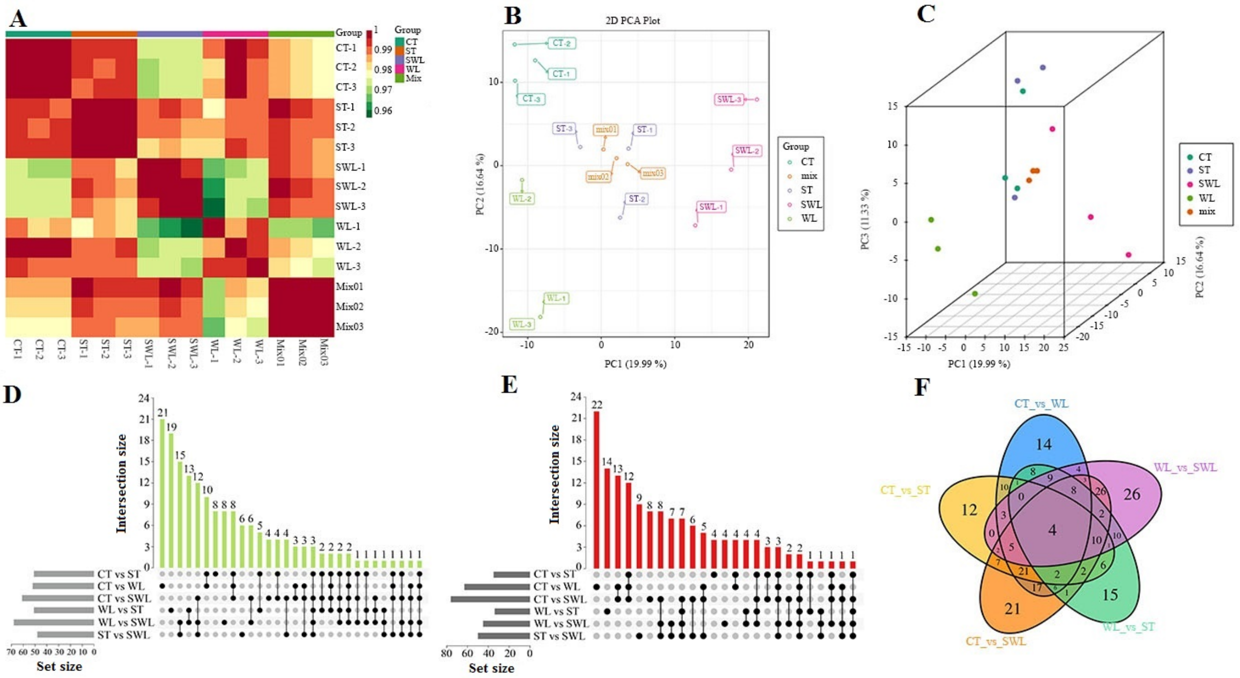

Supplemental Figure S1. Overview of the metabolomic data assessment in response to single and combined stress. Metabolomic of all samples clustering heatmap, various colours are obtained after normalization (red means high content, green means low content) (**A**). 2D plot of the principal component analysis (PCA) of the metabolites data set regrouping all treatments (**B**) and 3D plot (**C**). Intersections among down- (**D**) and up-regulated (**E**) differentially expressed metabolites across single and combined stresses. Number above bars showed the number of metabolites within each intersection. Venn diagrams display the relationship among different metabolites in various comparative groups, including CT, ST, WL, and SWL (**F**). Treatments are presented as follows: control (CT), salinity (ST), waterlogging (WL), and combination of WL and ST (SWL).

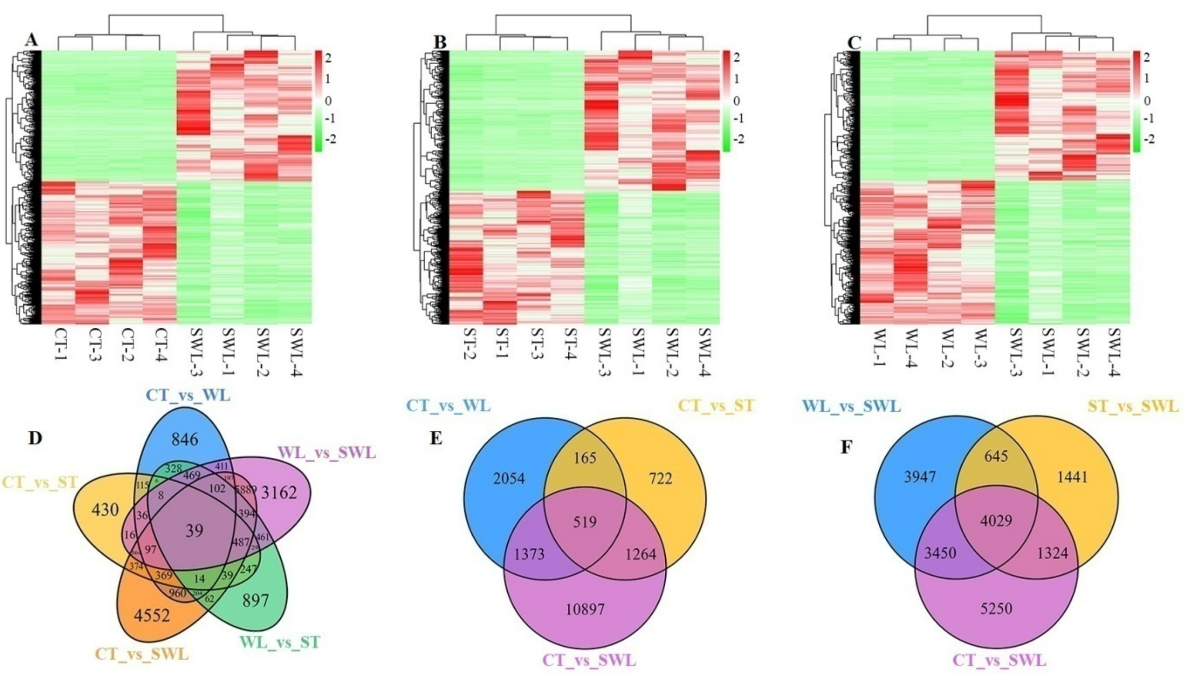

Supplemental Figure S2. Summary of the transcriptomic data assessment in response to single and combined stress. Cluster heat map analysis of expressed genes among CT vs SWL (**A**) ST vs SWL (**B**) and WL vs SWL (**C**). Interactive Venn diagram of differential gene results for different groups, overlapping areas indicate intersections between various combinatory groups, and numbers represent the number of differential genes (**D**, **E** and **F**). Treatments are presented as follows: control (CT), salinity (ST), waterlogging (WL), and combination of WL and ST (SWL).

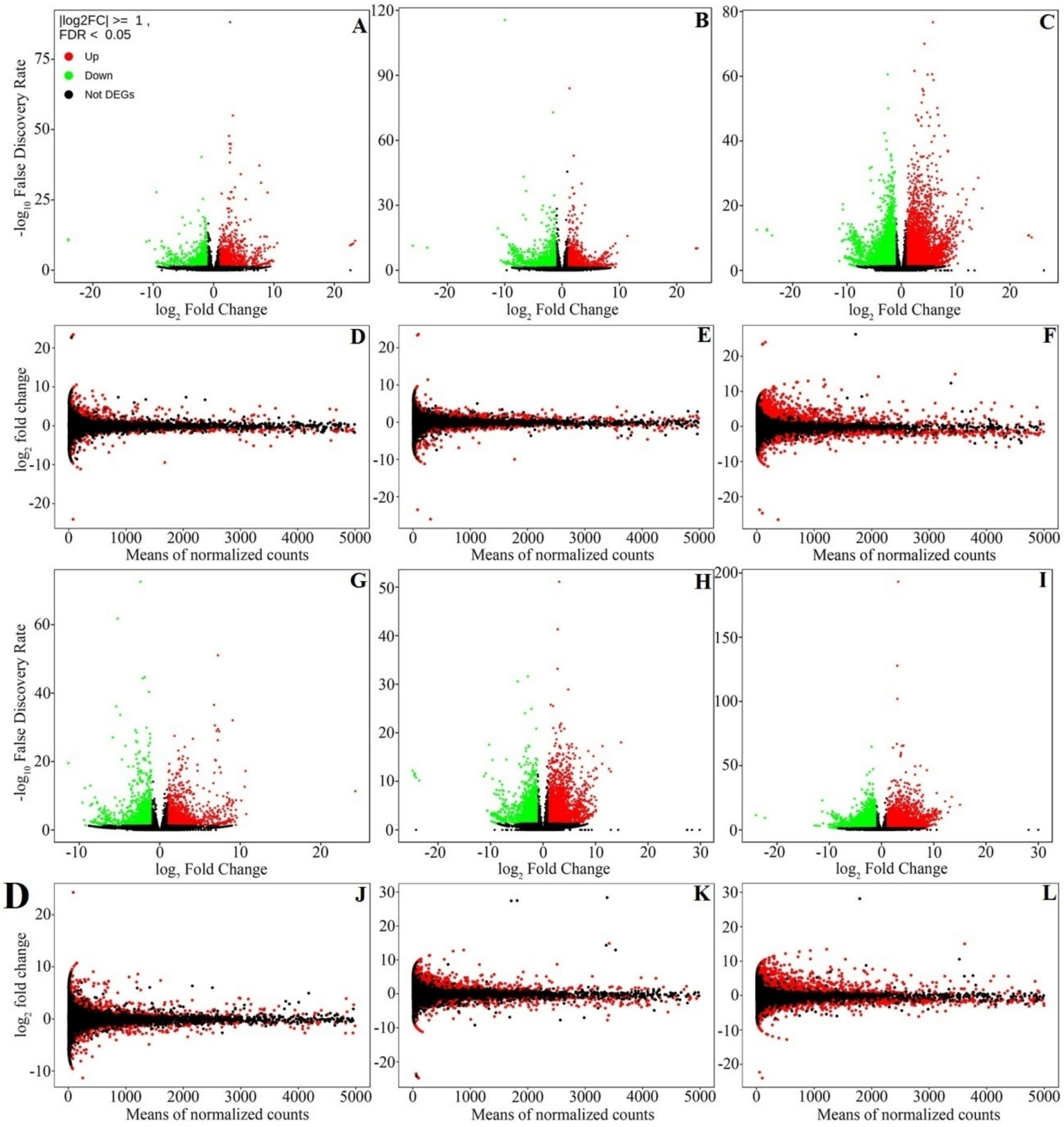

Supplemental Figure S3. Volcano plots and MA-plots of DEGs from fragrant rosewood under single and combined stress. Volcano plots of DEGs from ST, WL and SWL (**A, B and C)** treatments compared to CT based on the log2FC and the false detection rate (FDR < 0.05), the green colour point corresponded to the down-regulated genes and red colour those up-regulated. MA-plots of DEGs from ST, WL and SWL (**D, E and F**) treatments compared to CT, transcripts will be coloured in red if the FDR is less than 0.05 and the rest of DEGs were coloured in black. Volcano plots of DEGs from SWL compared to WL and ST and from ST compared to WL (**G, H and I**). MA-plots of DEGs from SWL compared to WL and ST and from ST compared to WL (**J, K and L**). Treatments are presented as follows: control (CT), salinity (ST), waterlogging (WL), and combination of WL and ST (SWL).

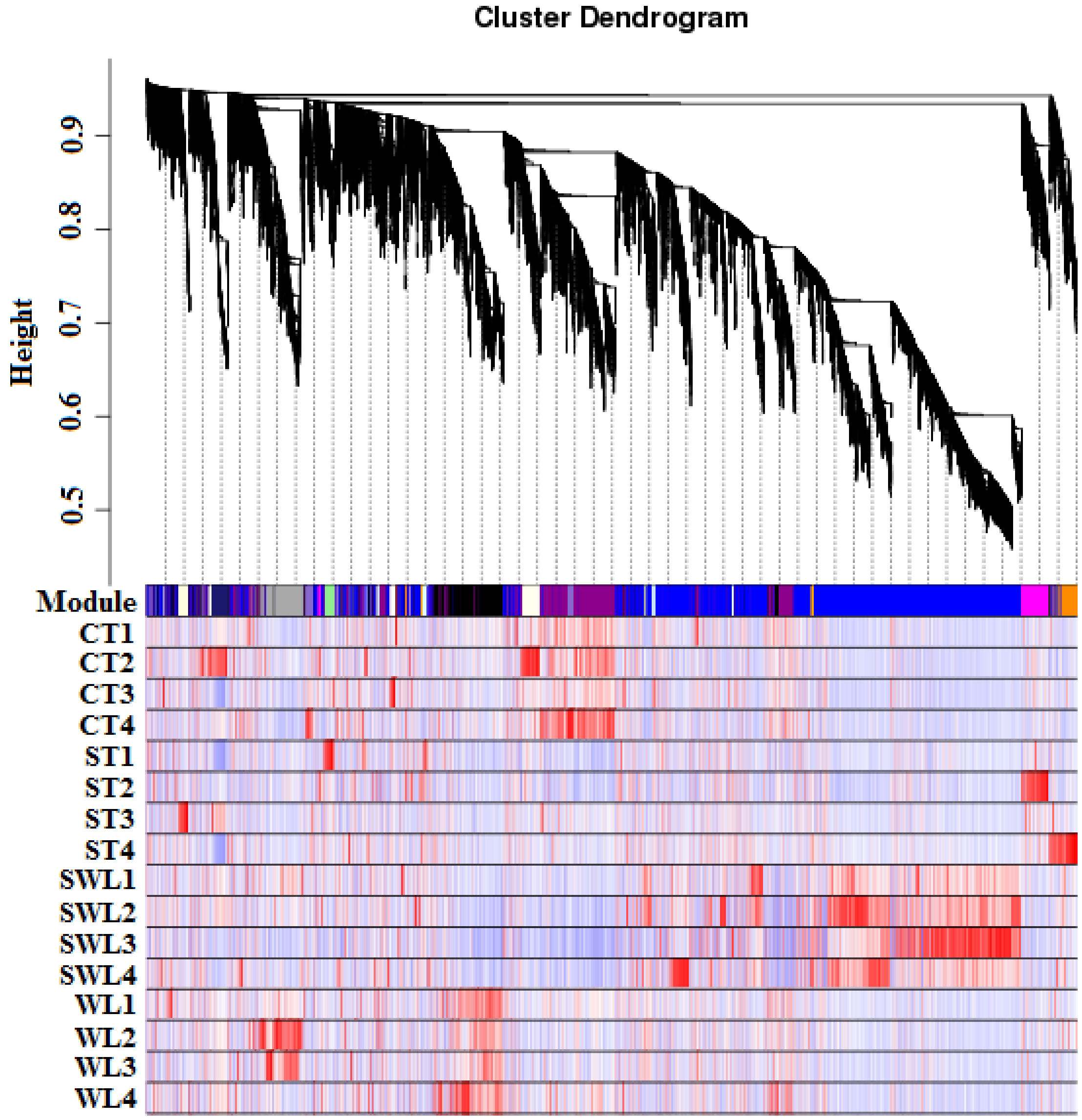

Supplemental Figure S4. Weighted gene co-expression network analysis cluster dendrogram among waterlogging, salinity and waterlogging combined with salinity in fragrant rosewood. Treatments are presented as follows: control (CT), salinity (ST), waterlogging (WL), and combination of WL and ST (SWL). The module color were: black, blue, brown 4, dark grey, dark magenta, dark orange, dark orange 2, ivory, light cyan 1, light green, light steel blue 1, light yellow, magenta, medium purple 3, mid night blue, orange and white.

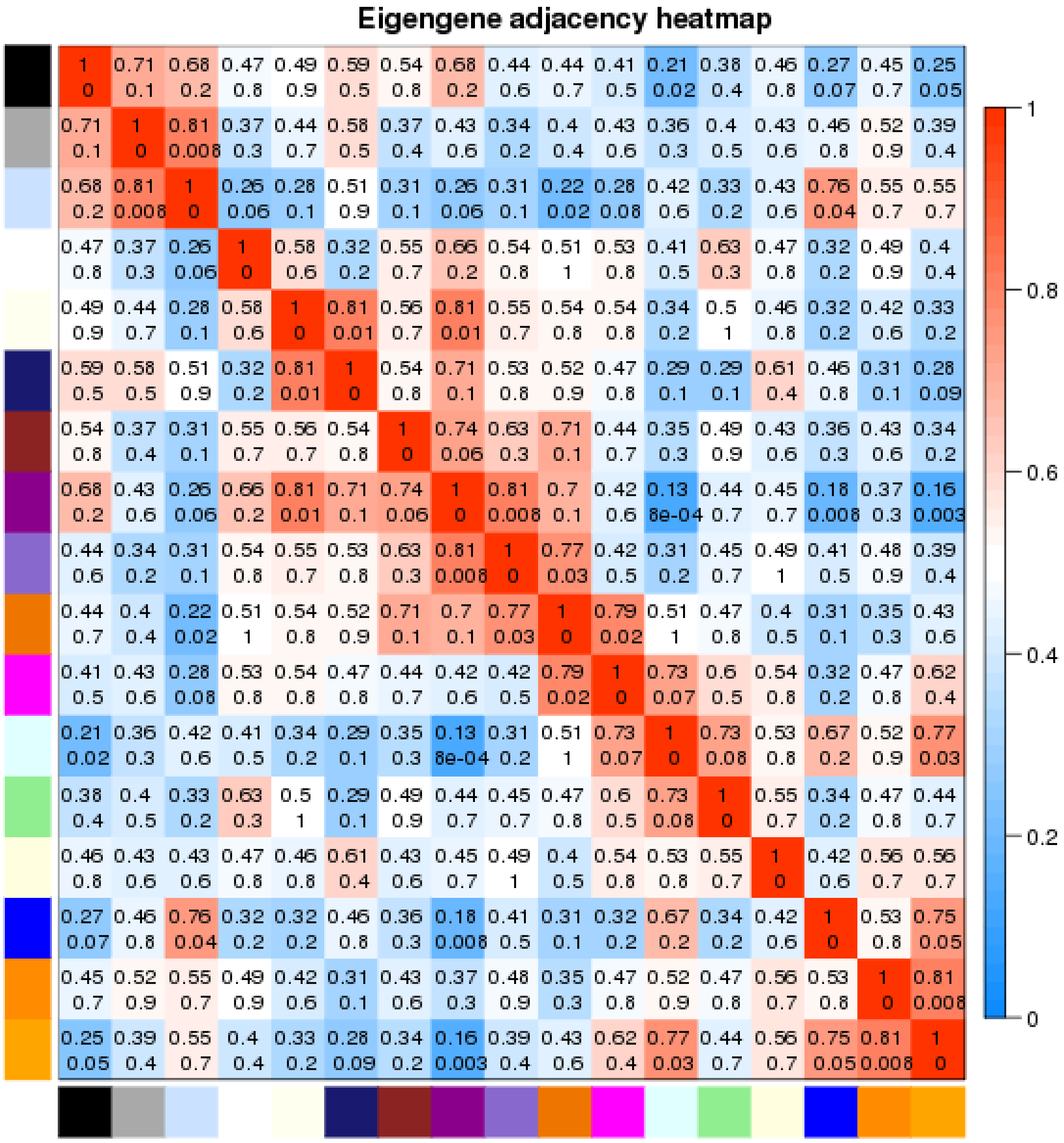

Supplemental Figure S5. Hub gene heatmap of the weighted gene co-expression network analysis (Eigengene adjacency).

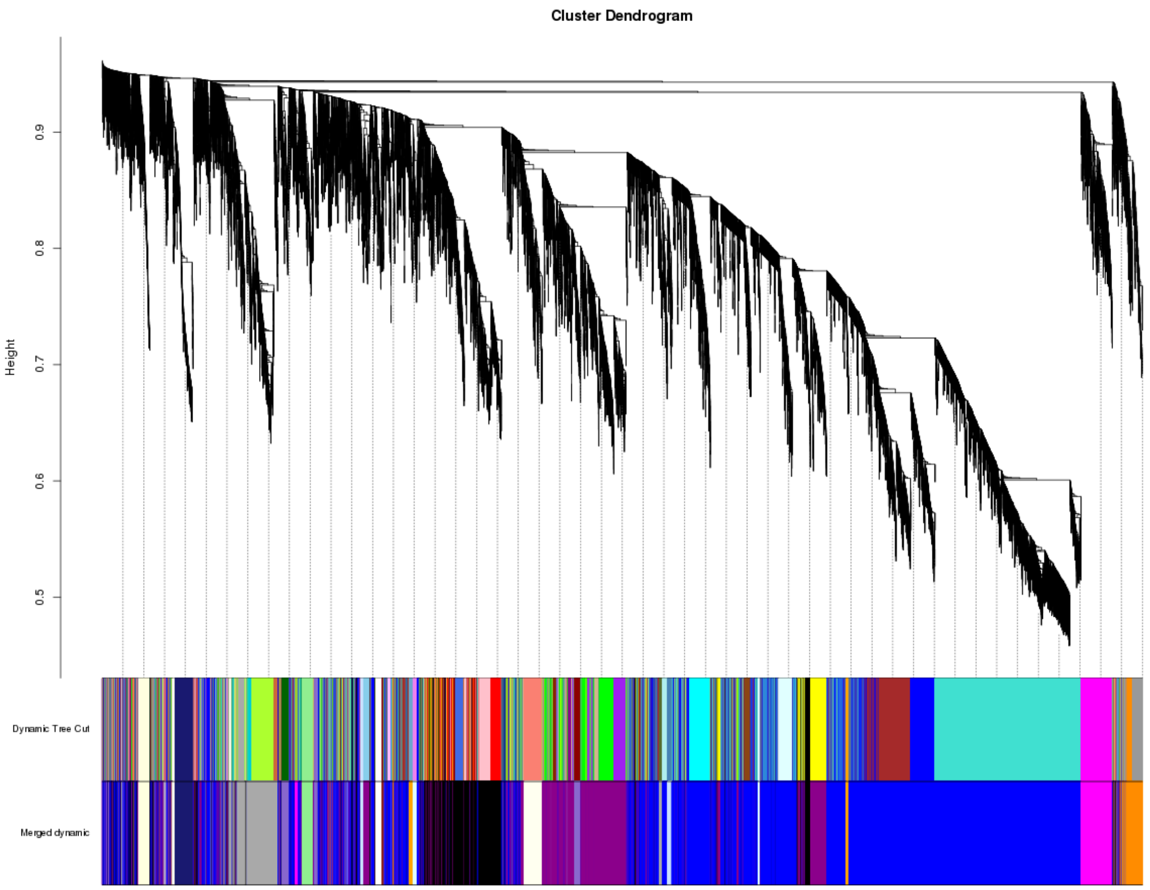

Supplemental Figure S6. Merged module tree of the weighted gene co-expression network analysis. The module color were: black, blue, brown 4, dark grey, dark magenta, dark orange, dark orange 2, ivory, light cyan 1, light green, light steel blue 1, light yellow, magenta, medium purple 3, mid night blue, orange and white.

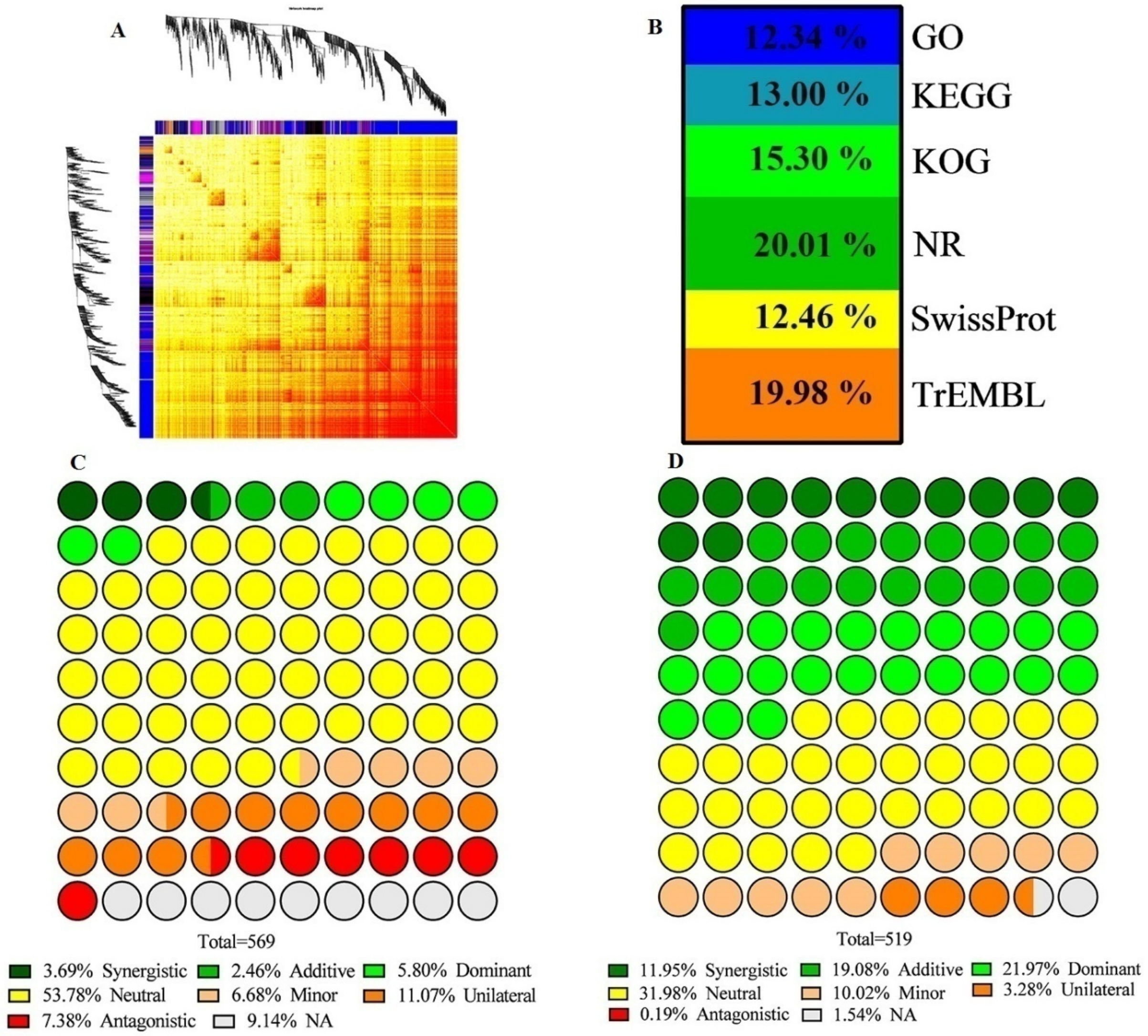

Supplemental Figure S7. Network heat map of the weighted gene co-expression network analysis and the different pattern mode found in fragrant rosewood under single and combined stress. Network heat map of the weighted gene co-expression network analysis (**A**). The Percentage of transcriptome annotation belonged to each database used in this study (**B**). Metabolomic and transcriptional patterns based on log2FC, regulation status (down or up) and standard deviation (SD ≤ 0.50) of *Dalbergia odorifera* responses to single and combined stress (synergistic, additive, dominant, neutral, minor, unilateral, and antagonistic). 10 x 10 dot plot showing the pattern modes; in SWL of the whole metabolites detected (569 metabolites) (**C**) and in the common differentially expressed transcript (519) (**D**).

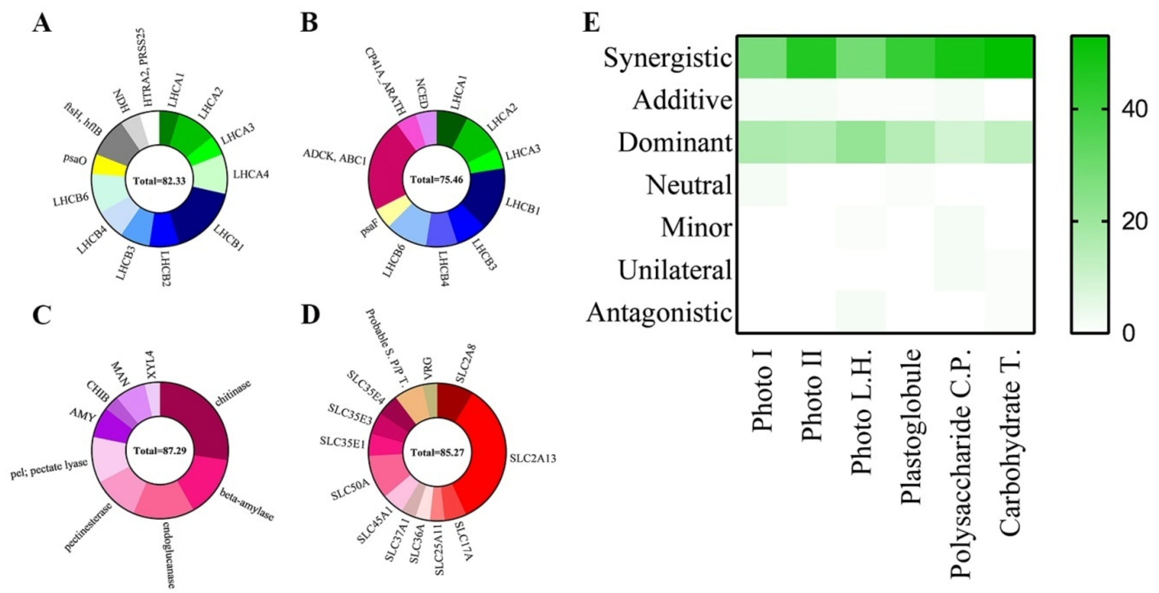

Supplemental Figure S8. Donut-plots and heat map of different functional GO classifications related to DEGs belong to photosynthesis and carbon metabolism. Donut-plots regrouping photosynthesis light harvesting (**A**) plastoglobule (**B**) polysaccharide catabolic process (**C**) and carbohydrate transport (**D**) related genes and their isoforms based on gene ontology (GO) classification and their relative frequency (ω). Heat map of the pattern modes in SWL based on functional GO classification related-DEGs in photosystem I (Photo I), photosystem II (Photo II), photosynthesis light harvesting (Photo L.H.), plastoglobule, polysaccharide catabolic process (Polysaccharide C.P.) and carbohydrate transport (Carbohydrate T.) classes (**E**).

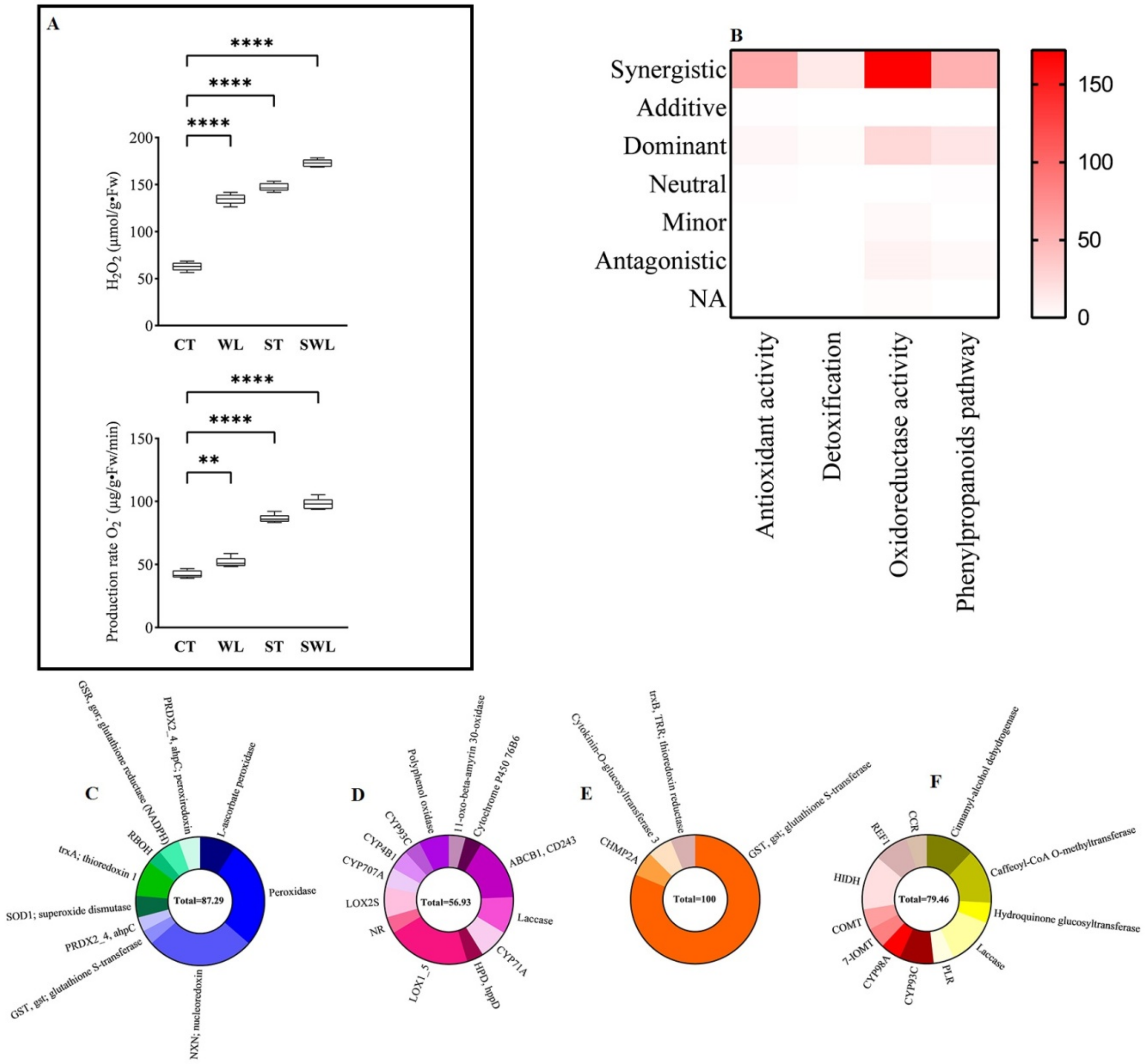

Supplemental Fig. S9. Oxidative stress indicators and functional GO classification related-DEGs in antioxidant activity, detoxification, oxido-reductase activity and phenylpropanoids pathway module. Reactive oxygen species (H_2O_2 and $O_2^{\bullet -}$) variations in *D. odorifera* under single and combined stress (**A**). Heat map of the pattern modes in SWL based on functional GO classification related-DEGs in antioxidant activity, detoxification, oxido-reductase activity and phenylpropanoids pathway classes (**B**). Donut-plots regrouping antioxidant activity (**C**) detoxification (**D**) oxido-reductase activity (**E**) and phenylpropanoids pathway (**F**) related genes and their isoforms based on GO classification and their relative frequency (ω). Treatments are presented as follows: control (CT), salinity (ST), waterlogging (WL), and combination of WL and ST (SWL). Statistical analysis was performed with one way ANOVA with Tukey's honestly significant difference test (Graph Pad prism 9.0.0). Data were expressed as means ± SD (5 replicates from 5 different seedlings), and significant differences between means were determined at a p-value ≤ 0.05. The perpendicular line within each box

represents the median value and the ends of the box represent the 3rd and 1st quartile. **; $P < 0.002$, ****; $P < 0.0001$

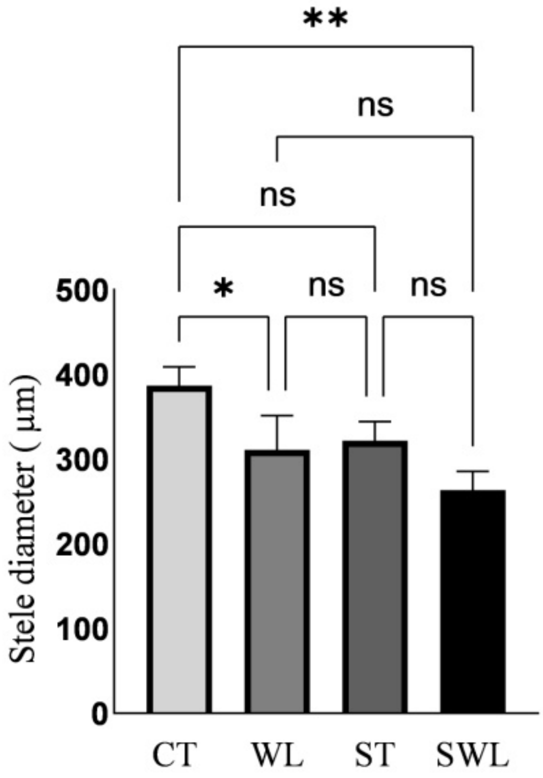

Supplemental Figure S10. Average of root stele diameter variations in *D. odorifera* seedlings under CT, WL, ST and SWL. Treatments are presented as follows: control (CT), salinity (ST), waterlogging (WL), and combination of WL and ST (SWL). The signs such as *, **, and NS represented the significance difference among different treatments. *; P < 0.05, **; P < 0.002, NS; no significant. Statistical analysis was performed with one way ANOVA with Tukey's honestly significant difference test (Graph Pad prism 9.0.0). Data are expressed as mean ± standard deviation and the symptoms were observed in five replicates from 5 different seedlings.

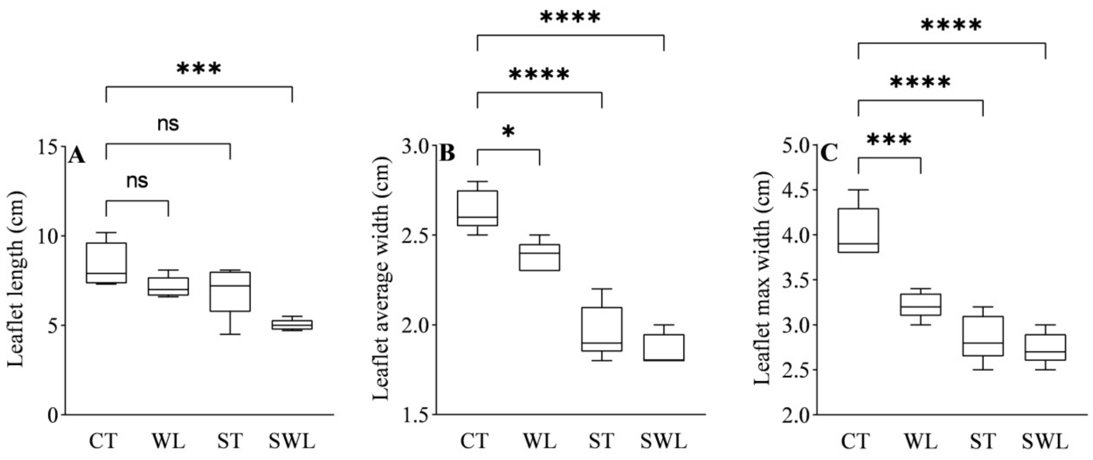

Supplemental Figure S11. Leaf morphology variations in fragrant rosewood leaflets under single and combined stress. Leaf length (**A**), leaflet average width (**B**) and leaflet max width (**C**) variations in *D. odorifera* leaflets under CT, WL, ST and SWL. Treatments are presented as follows: control (CT), salinity (ST), waterlogging (WL), and combination of WL and ST (SWL). The signs such as *, ***, **** and NS represented the significance difference among different treatments. Statistical analysis was performed with one way ANOVA with Tukey's honestly significant difference test (Graph Pad prism 9.0.0). Data were expressed as means ± SD (5 replicates from 5 different seedlings), and significant differences between means were determined at a p-value ≤ 0.05. The perpendicular line within each box represents the median value and the ends of the box represent the 3rd and 1st quartile. *; $P < 0.05$, ***; $P = 0.0001$, ****; $P < 0.0001$, NS; no significant.

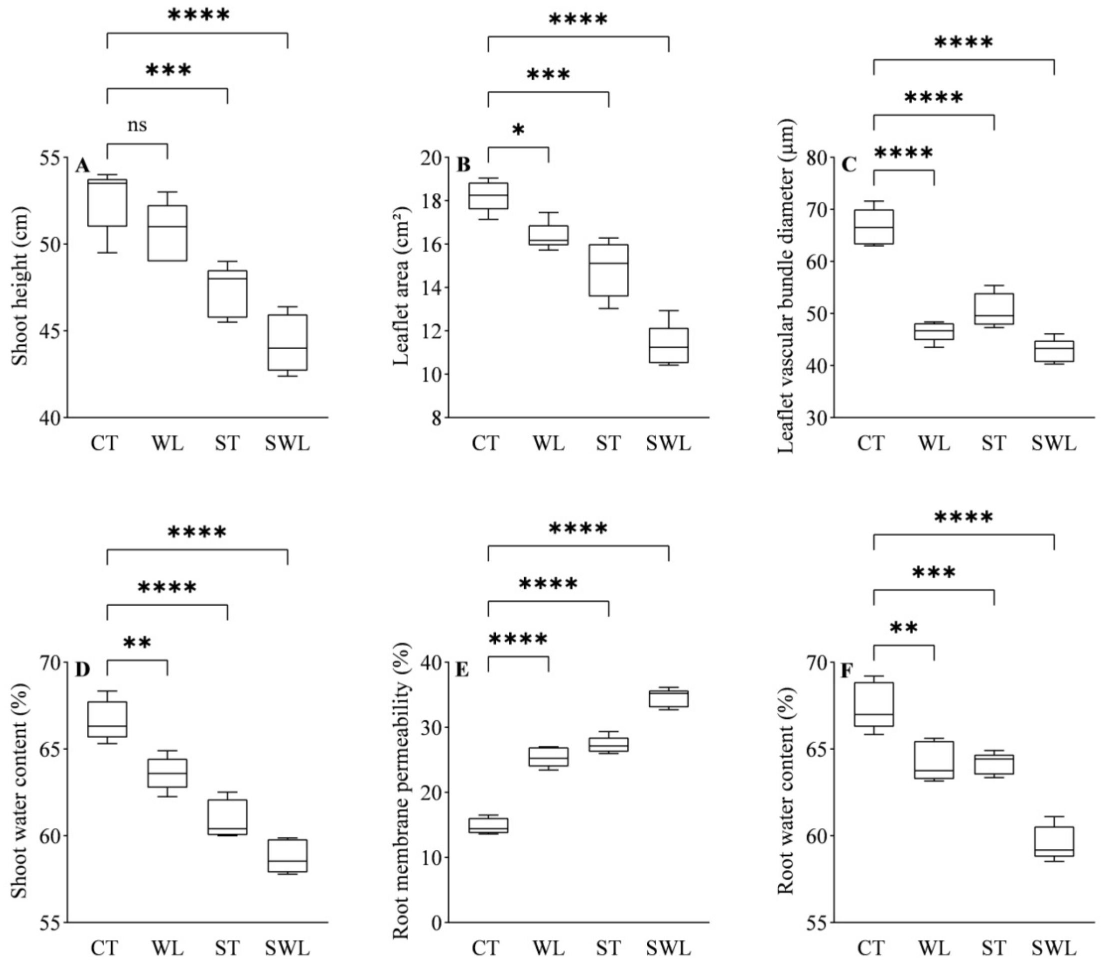

Supplemental Figure S12. Leaf and/or root anatomy, morphology and water status in fragrant rosewood seedlings under single and combined stress. Variation in shoot height (**A**), leaflet area (**B**), vascular bundle diameter (**C**), shoot water content (**D**), root membrane permeability (**E**) and root water content (**F**) in *Dalbergia odorifera* seedlings under CT, WL, ST and SWL. Treatments are presented as follows: control (CT), salinity (ST), waterlogging (WL), and combination of WL and ST (SWL). The signs such as *, ***, **** and NS represented the significance difference among different treatments. Statistical analysis was performed with one way ANOVA with Tukey's honestly significant difference test (Graph Pad prism 9.0.0). Data were expressed as means ± SD (5 replicates from 5 different seedlings), and significant differences between means were determined at a p-value ≤ 0.05. The perpendicular line within each box represents the median value and the ends of the box represent the 3rd and 1st quartile. *; P < 0.05, ***; P = 0.0001, ****; P < 0.0001, NS; no significant.

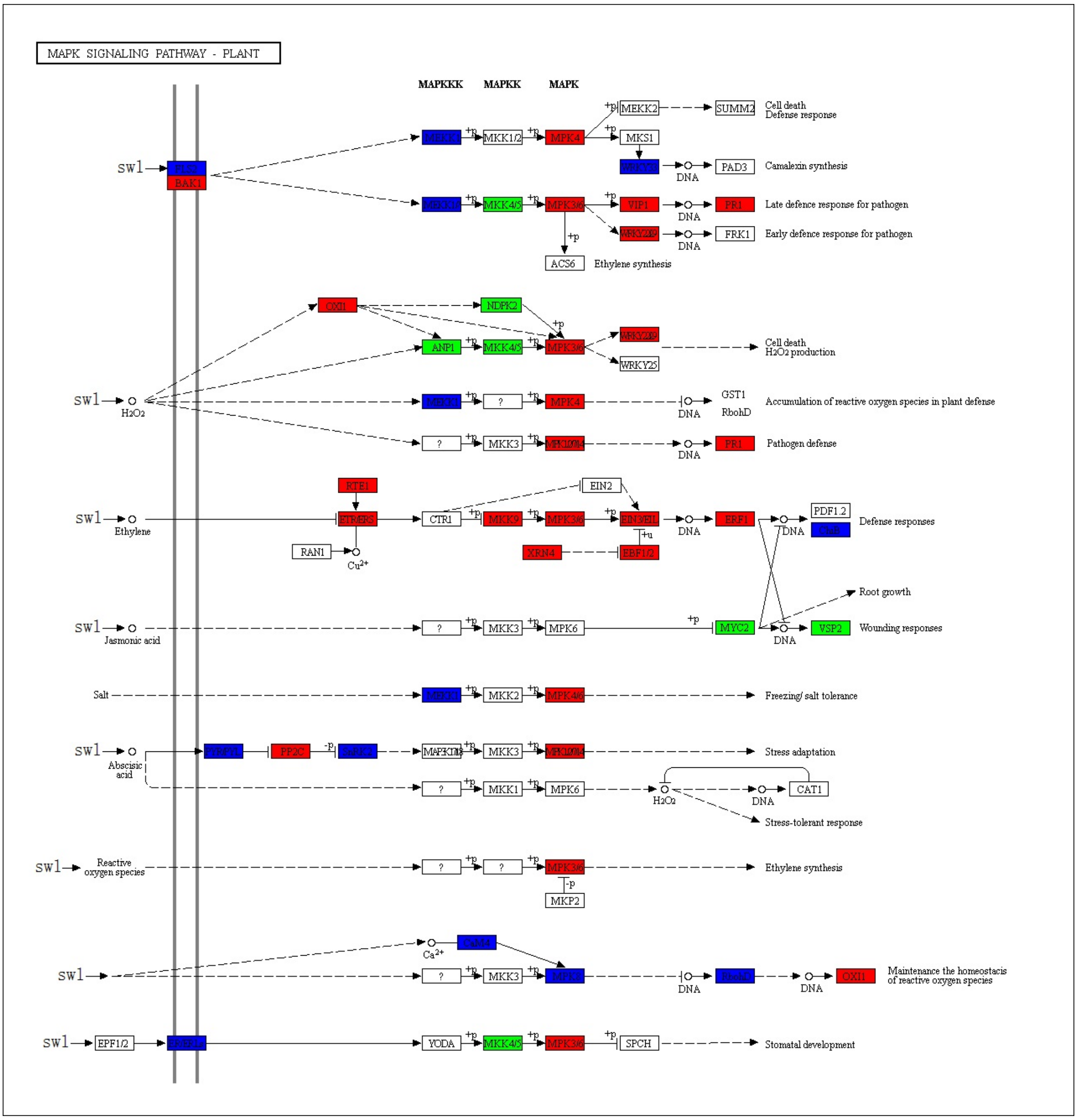

Supplemental Figure S13. KEGG of plant MAPK signaling pathway in fragrant rosewood leaflet under combined stress. KO nodes containing only up-regulated differential genes are marked in red, KO nodes containing only down-regulated differential genes are marked in green, and KO nodes containing both up-regulated and down-regulated genes are marked in blue.

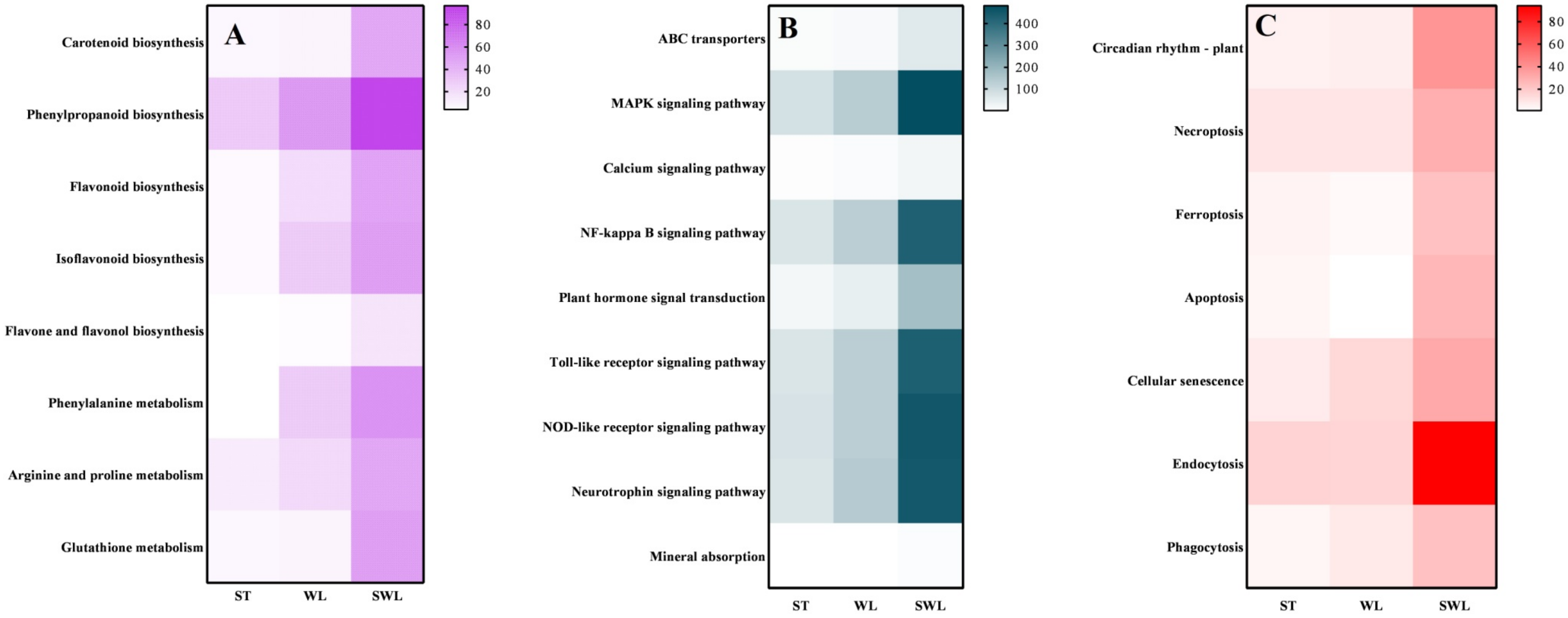

Supplemental Figure S14. Heatmap regrouping differentially genes enrichment following gene ontology biological function, the scale represents the number of transcripts and isoforms of each genes following GO classification. Transcripts involved in phenylpropanoids pathway, proline and glutathione metabolism (**A**), transcripts involved in mineral absorption, ABC transporters and signaling pathway (**B**) and those related to plant cellular death, senescence and the circadian rhythm (**C**).

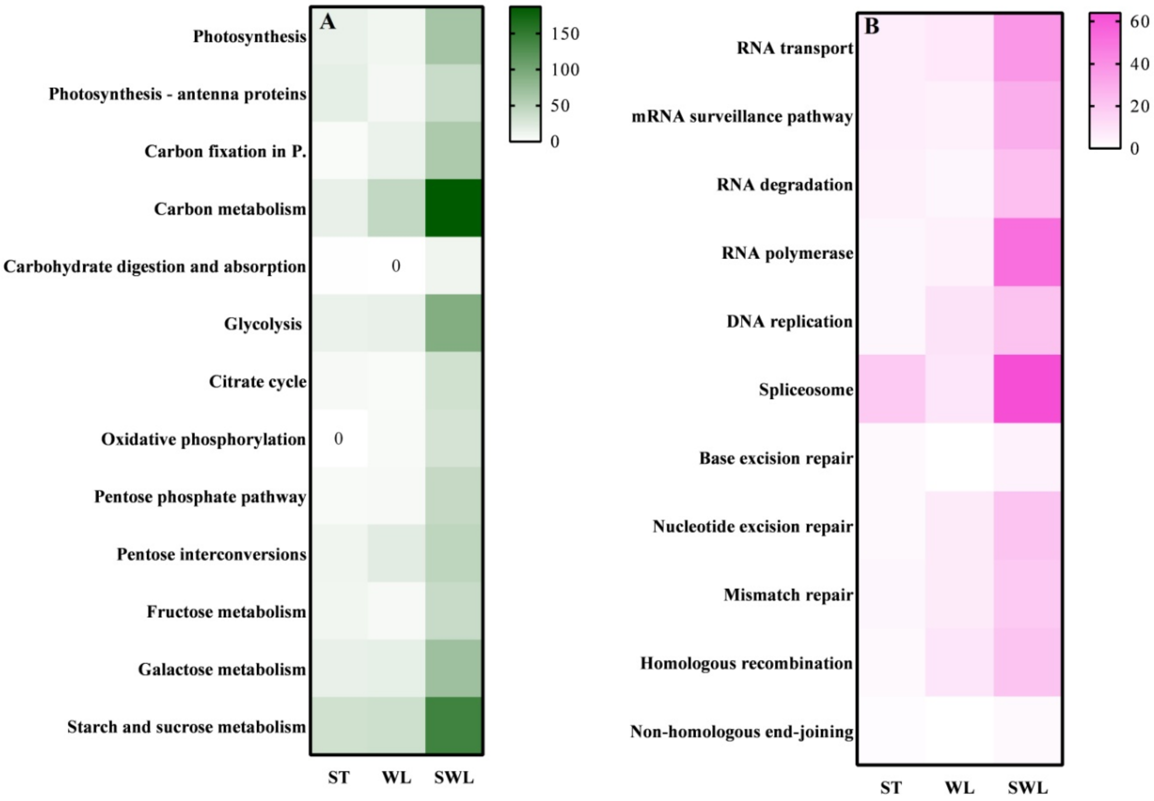

Supplemental Figure S15. Heatmap regrouping differentially genes enrichment following gene ontology biological function related to DNA, RNA activities and other modules. the scale represents the number of transcripts and isoforms of each genes following GO classification. Transcripts involved in phenylpropanoids pathway, proline and glutathione metabolism (**A**), transcripts related to photosynthesis (**B**) and those related to DNA and RNA activities.

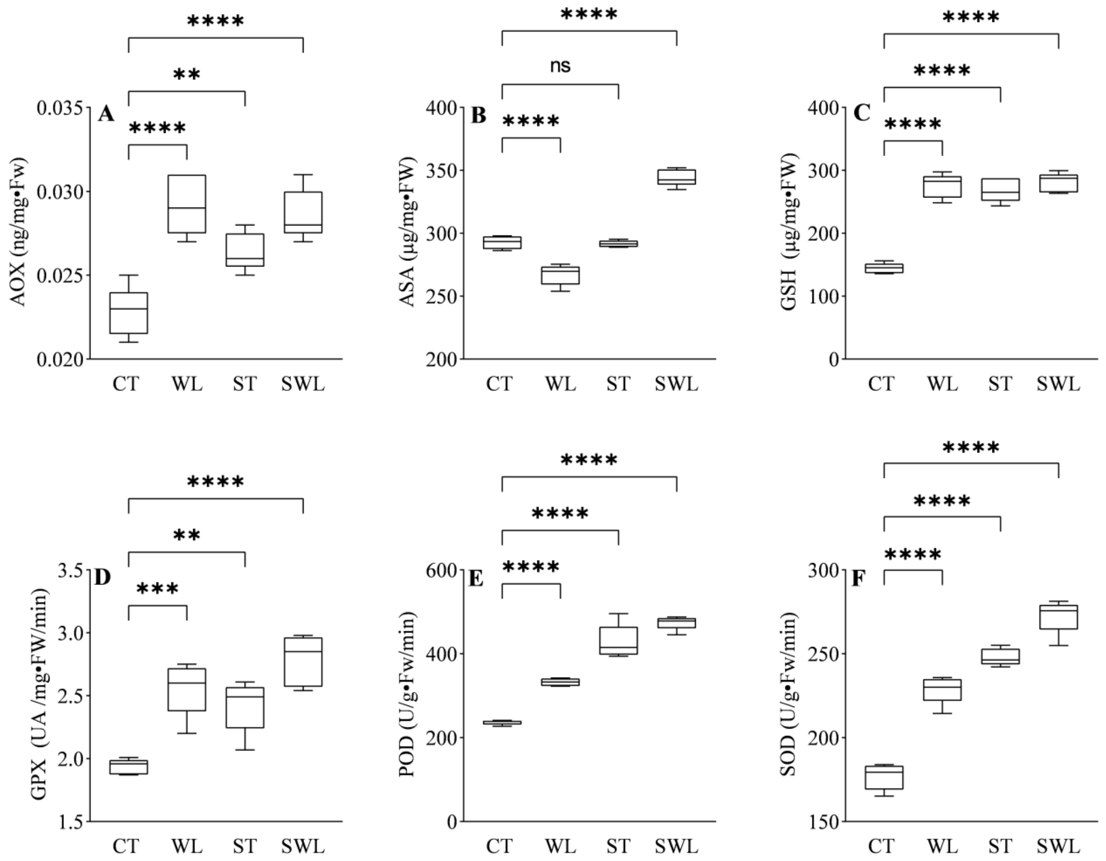

Supplemental Figure S16. Antioxidative enzymes and molecules variations in fragrant rosewood under single and combined stress. Variations in alternative oxidase (AOX) proteins (**A**), ascorbic acid (ASA) (**B**), and reduced glutathione (GSH) (**C**) contents, glutathione peroxidase (GPX) (**D**), peroxidases (POD) (**E**), and superoxide dismutase (SOD) (**F**) activities in *D. odorifera* leaflets under CT, WL, ST and SWL. Treatments are presented as follows: control (CT), salinity (ST), waterlogging (WL), and combination of WL and ST (SWL). The signs such as **, ***, **** and NS represented the significance difference among different treatments. Statistical analysis was performed with one way ANOVA with Tukey's honestly significant difference test (Graph Pad prism 9.0.0). Data were expressed as means ± SD (5 replicates from 5 different seedlings), and significant differences between means were determined at a p-value ≤ 0.05. The perpendicular line within each box represents the median value and the ends of the box represent the 3rd and 1st quartile. **; P < 0.01, ***; P = 0.0001, ****; P < 0.0001, NS; no significant.

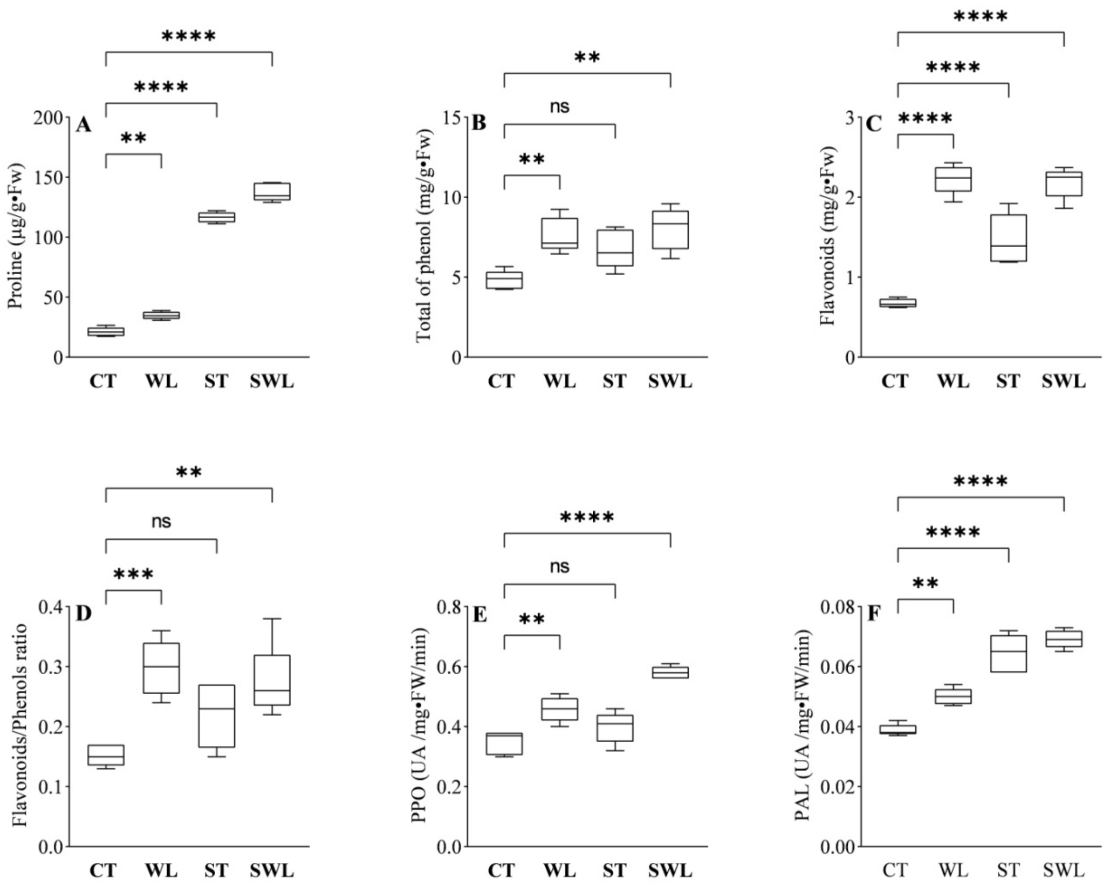

Supplemental Figure S17. Variations in Proline, total of phenol, and flavonoids in fragrant rosewood under single and combined stress. Variations in Proline (**A**), total of phenols (**B**), and flavonoids (**C**) contents, ration between flavonoids and total of phenols (**D**), polyphenol oxidase (PPO) (**E**) and phenylalanine ammonia-lyase (PAL) (**F**) activities in *D. odorifera* leaflets under CT, WL, ST and SWL. Treatments are presented as follows: control (CT), salinity (ST), waterlogging (WL), and combination of WL and ST (SWL). Statistical analysis was performed with one way ANOVA with Tukey's honestly significant difference test (Graph Pad prism 9.0.0). Data were expressed as means ± SD (5 replicates from 5 different seedlings), and significant differences between means were determined at a p-value ≤ 0.05. The perpendicular line within each box represents the median value and the ends of the box represent the 3rd and 1st quartile. The signs such as **, ***, **** and NS represented the significance difference among different treatments. **; P < 0.01, ***; P = 0.0001, ****; P < 0.0001, NS; no significant.

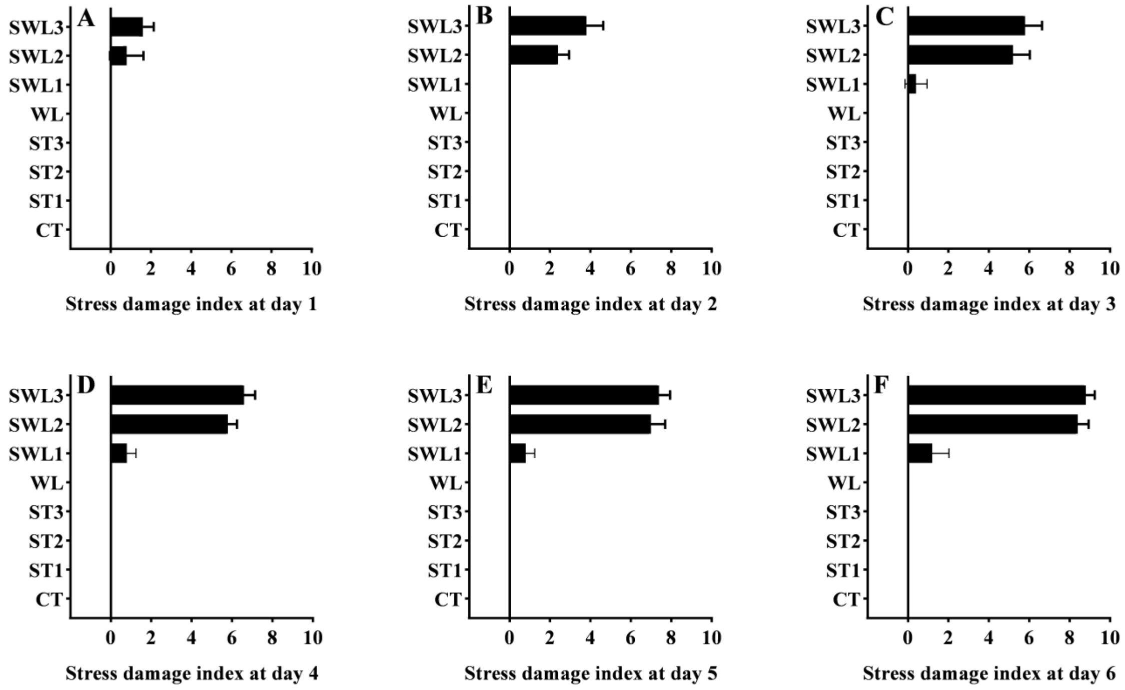

Supplemental Figure S18. Variations of stress damage index in *D. odorifera* leaflets under CT, WL, ST and SWL during the first experiment from day 1 to day 6. Treatments are presented as follows: control (CT), salinity (ST) (ST1, 100 mM; ST2, 150 mM; and 200 mM), waterlogging (WL), and combination of WL and ST (SWL) (SWL1, WL + ST1; SWL2, WL + ST2; and SWL3, WL + ST3). Data are expressed as mean ± standard deviation and the symptoms were observed in five replicates from 5 different seedlings.

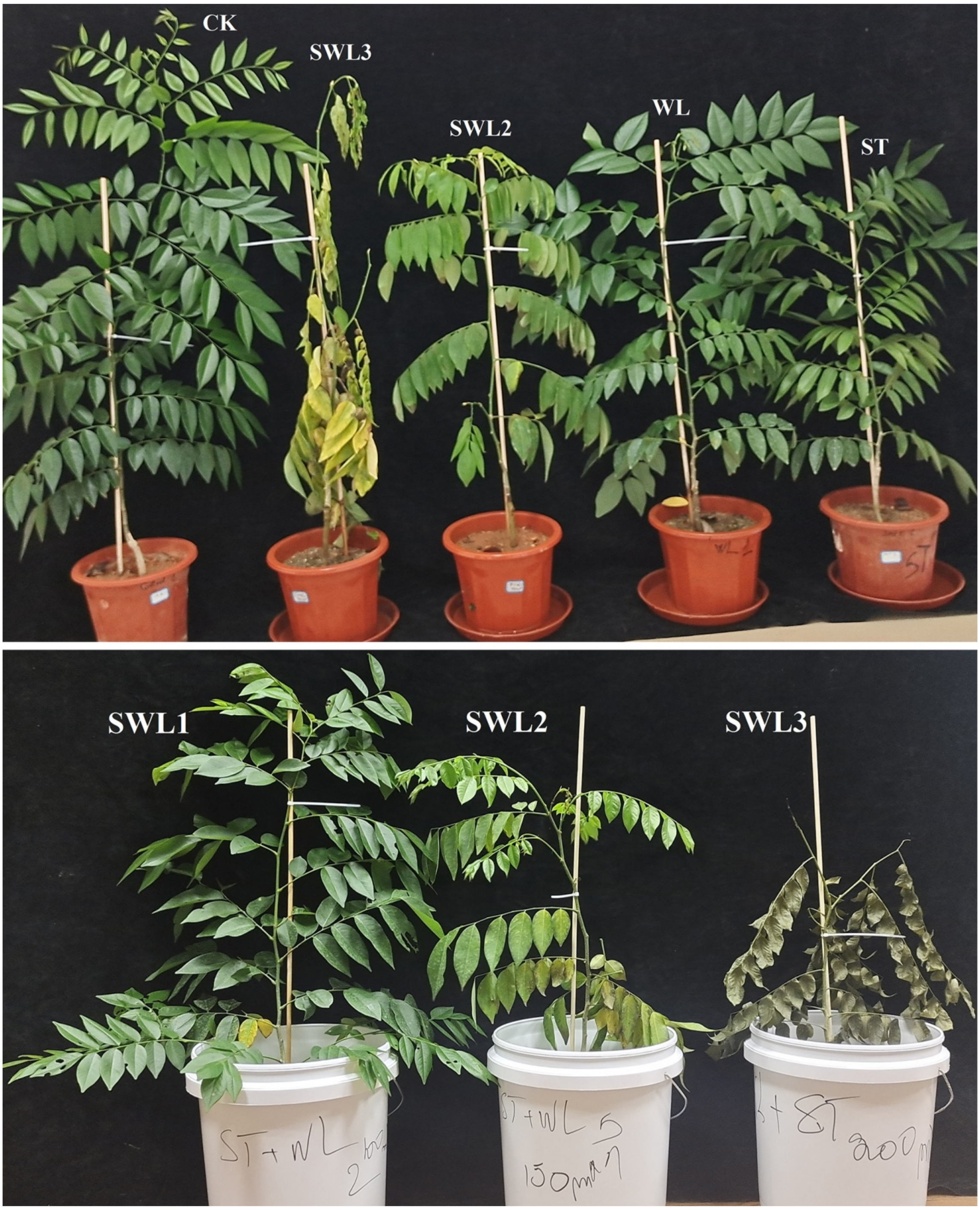

Supplemental Figure S19. Pictures of fragrant rosewood at day 6 under single and combined stress. Treatments are presented as follows: control (CK), salinity (ST) (ST1, 100 mM; ST2, 150 mM and ST3, 200 mM), waterlogging (WL), and combination of WL and ST (SWL) (SWL1, ST1 + WL; SWL2, WL + ST2 and SWL3, WL + ST3).

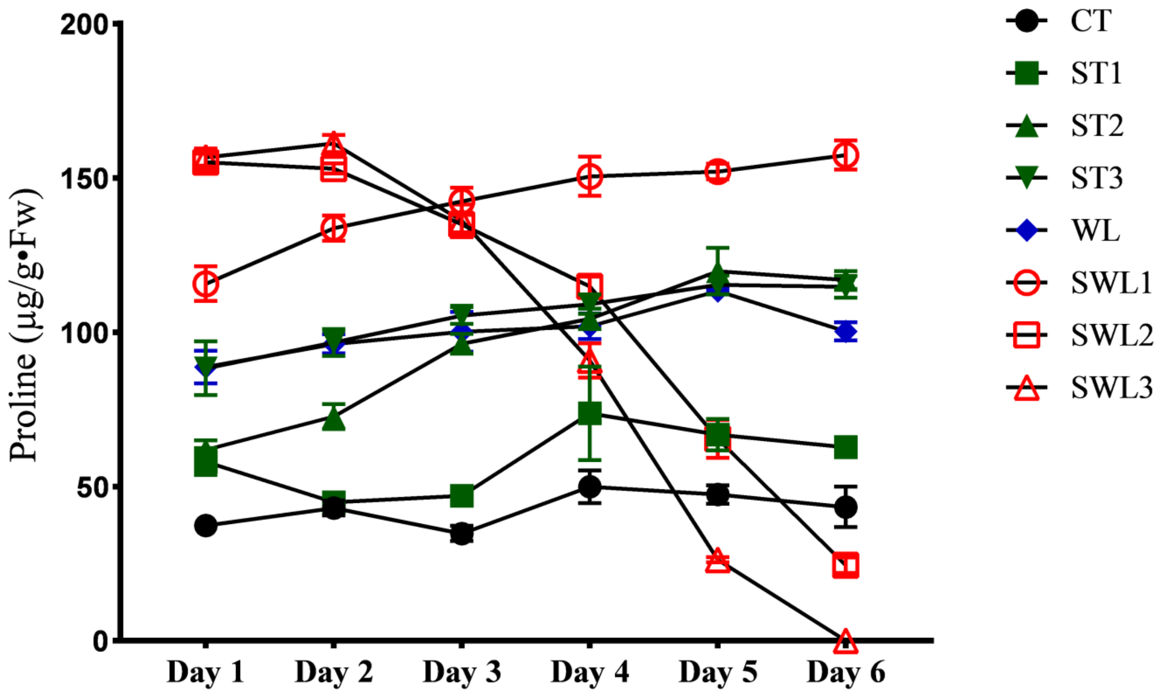

Supplemental Figure S20. Variations in proline content in fragrant rosewood under single and combined stress. Treatments are presented as follows: control (CT), salinity (ST) (ST1, 100 mM; ST2, 150 mM; and ST3, 200 mM), waterlogging (WL), and combination of WL and ST (SWL) (SWL1, WL + ST1; SWL2, WL + ST2; and SWL3, WL + ST3). Salinity at different concentrations (100 mM, 150 mM, or 200 mM) will induce proline as an adaptive response, and following the salt intensity, salt-induced damages will be non-significant, significant, or drastic which can lead to the death of the plant. To make sure that we triggered a significant number of salt-responsive metabolites and transcripts we have chosen 200 mM over 100 mM at day 6. Indeed, on day 6, 100 mM of salinity showed a proline level closer to CT compared to ST2 and ST3. Also, at day 6 SWL2 and SWL3 showed a dramatically low level of proline compared to CT. Thus SWL1 has been chosen over SWL2 and SWL3. Data are expressed as mean ± standard deviation and the symptoms were observed in five replicates from 5 different seedlings.

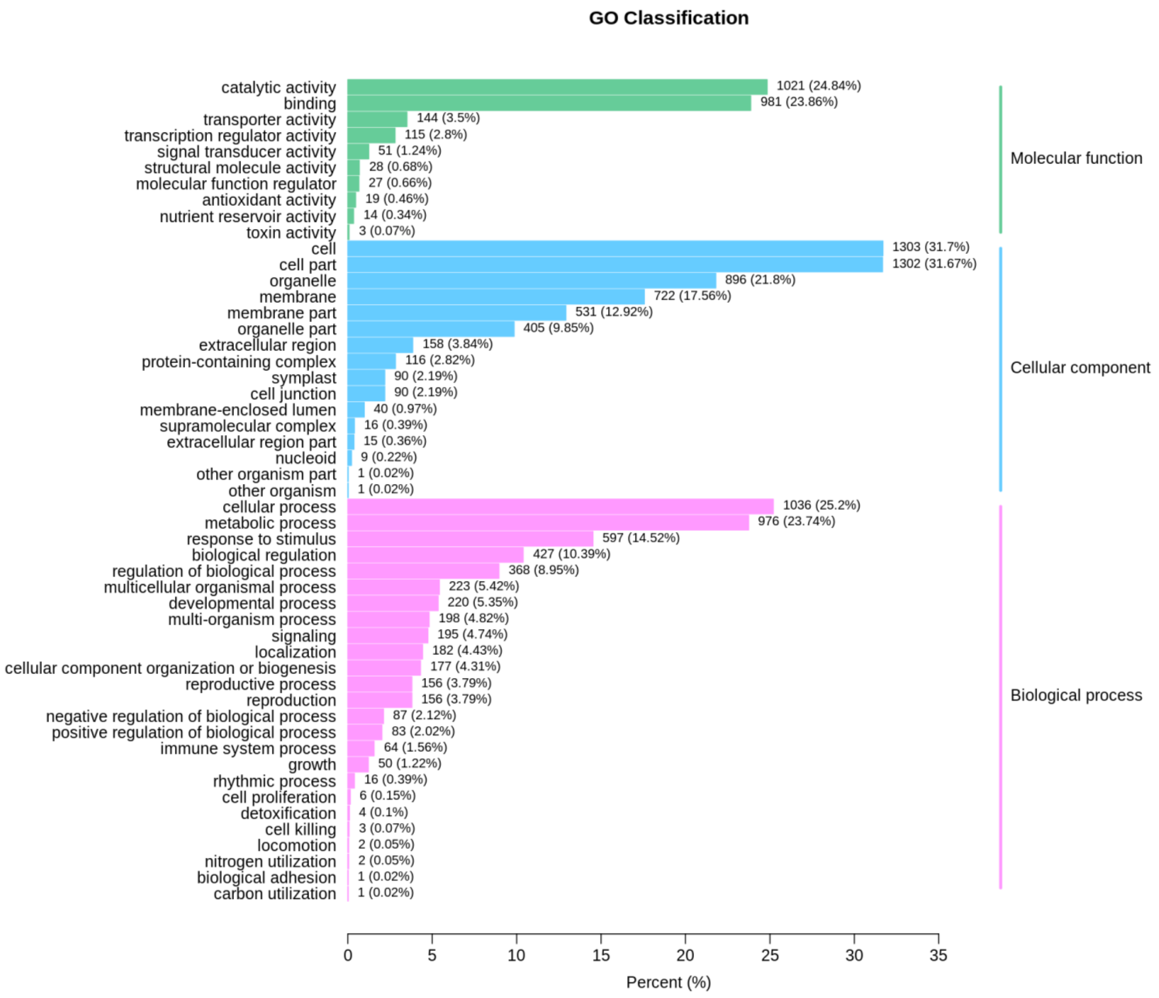

Supplemental Figure S21. GO classification for the differentially expressed genes in waterlogging-treated seedlings of fragrant rosewood. Following three categories: molecular function (green), cellular component (blue), and biological process (pink). X axis represents the percentage of DEGs and Y axis the GO terms.

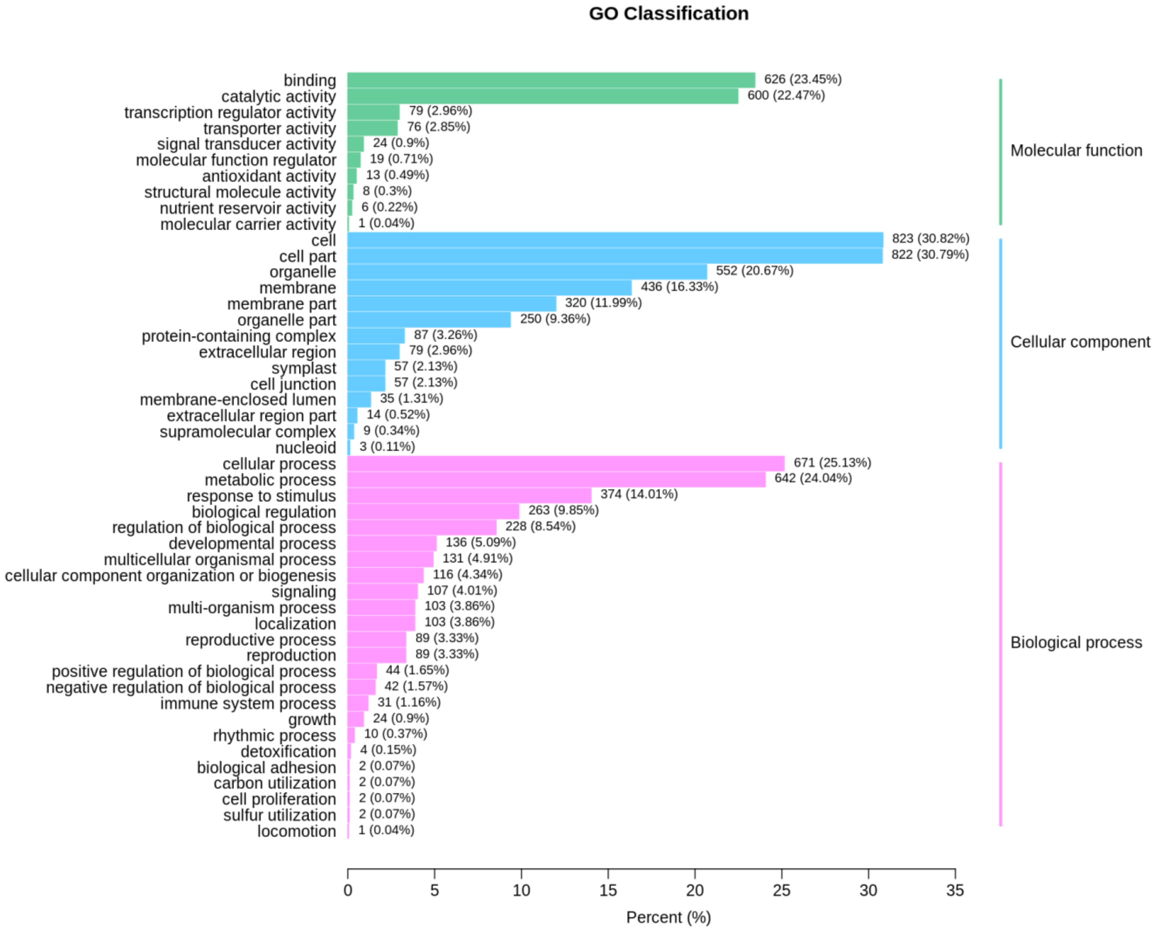

Supplemental Figure S22. GO classification for the differentially expressed genes in salinity-treated seedlings of fragrant rosewood. Following three categories: molecular function (green), cellular component (blue), and biological process (pink). X axis represents the percentage of DEGs and Y axis the GO terms.

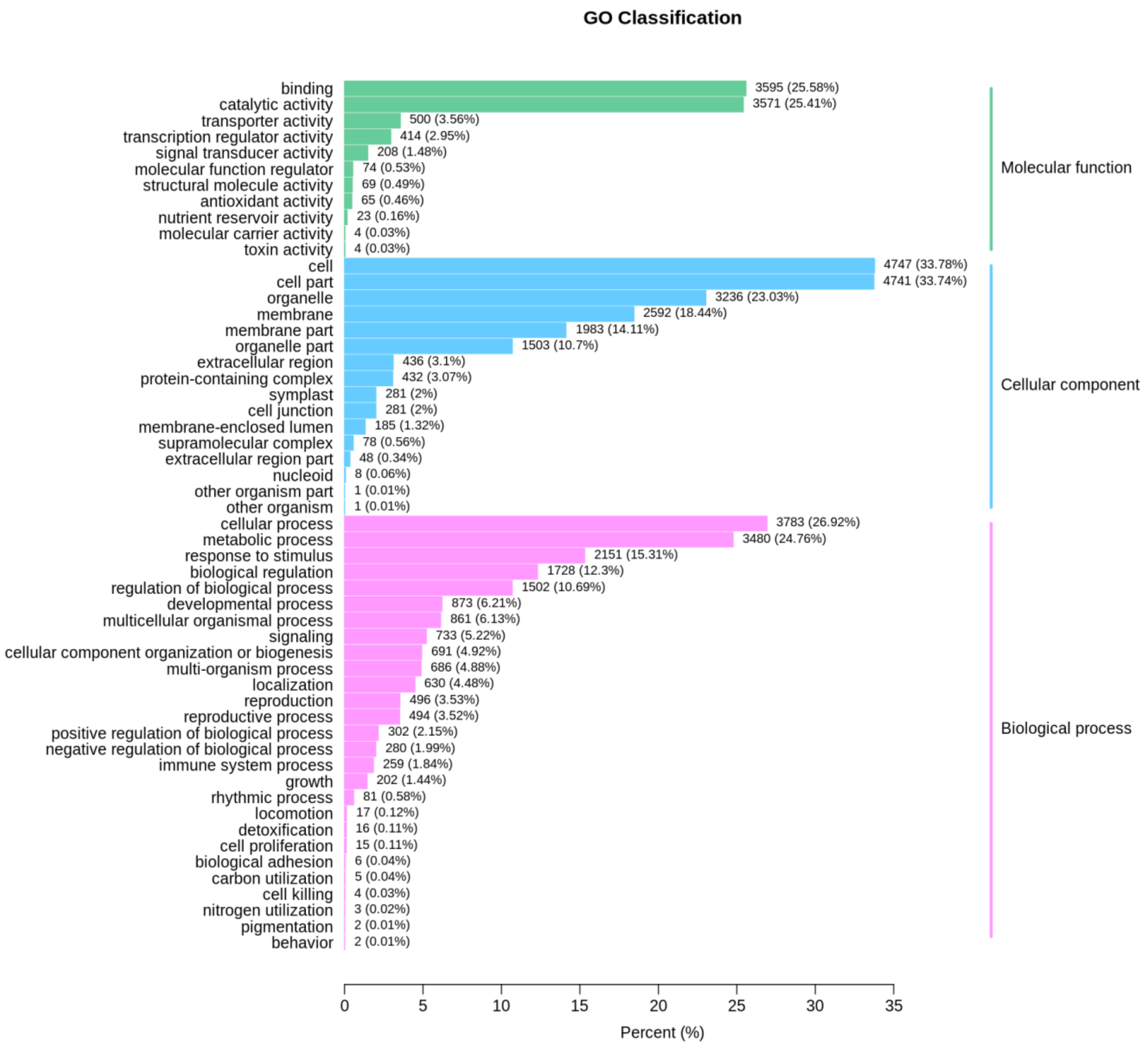

Supplemental Figure S23. GO classification for the differentially expressed genes in (salinity combined with waterlogging) SWL-treated seedlings of fragrant rosewood. Following three categories: molecular function (green), cellular component (blue), and biological process (pink). X axis represents the percentage of DEGs and Y axis the GO terms.

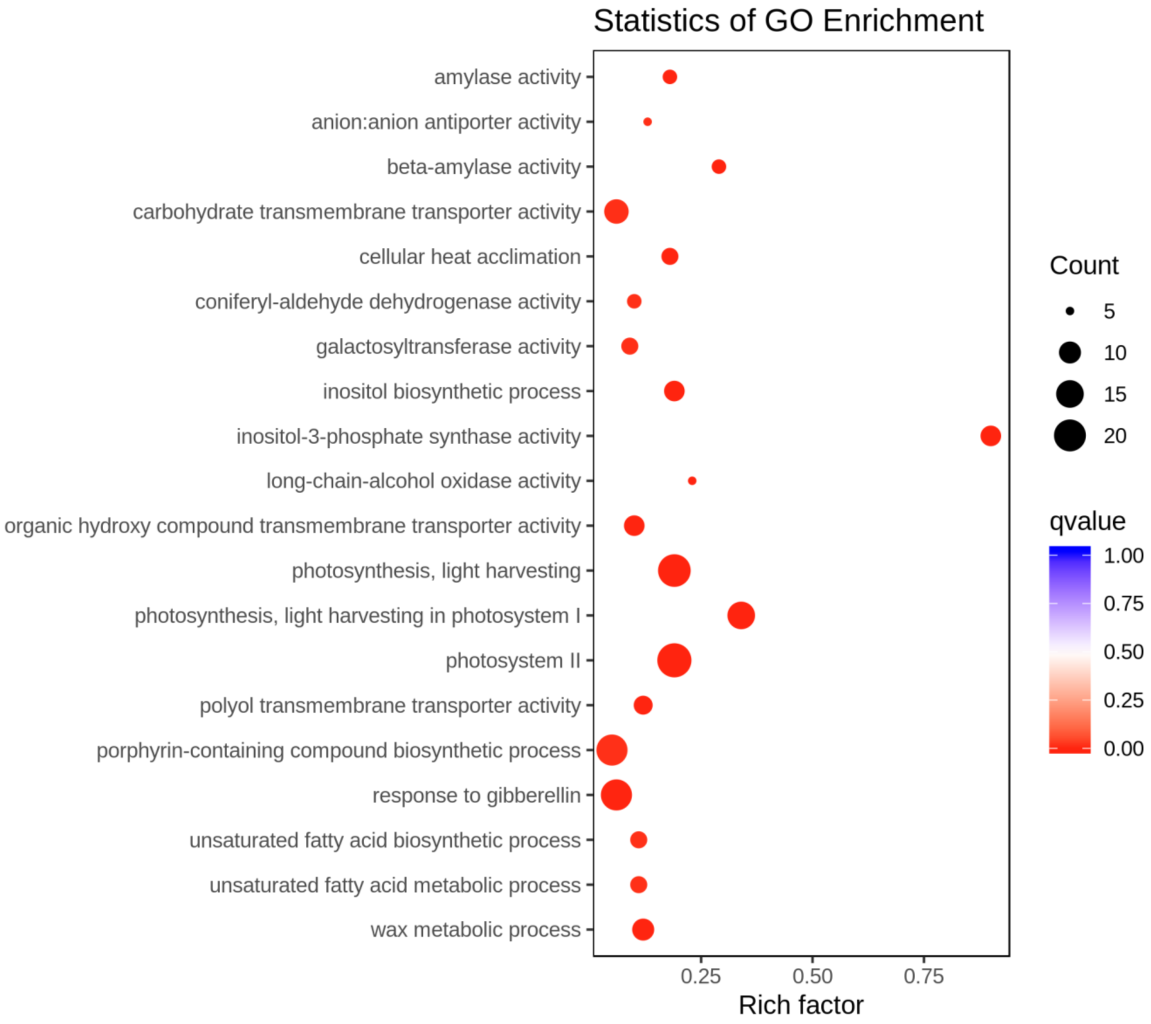

Supplemental Figure S24. GO enrichment scatter-plots of DEGs analysis between the control group versus salinity. Y axis indicates the KEGG pathway and X axis the rich factor the larger the rich factor, the greater the enrichment, the smaller the P value, and the more significant the enrichment.

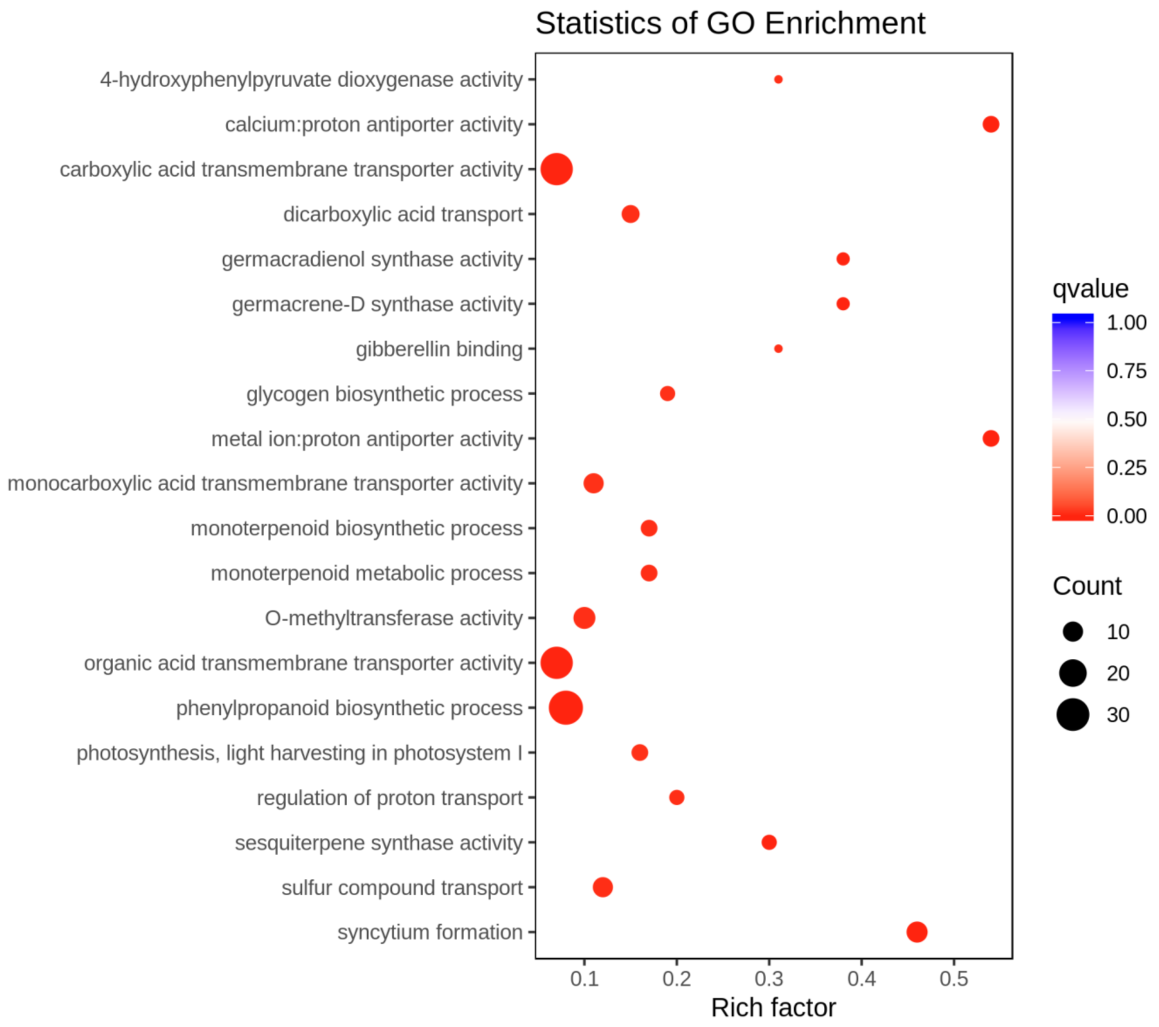

Supplemental Figure S25. GO enrichment scatter-plots of DEGs analysis between the control group versus waterlogging. Y axis indicates the KEGG pathway and X axis the rich factor the larger the rich factor, the greater the enrichment, the smaller the P value, and the more significant the enrichment.

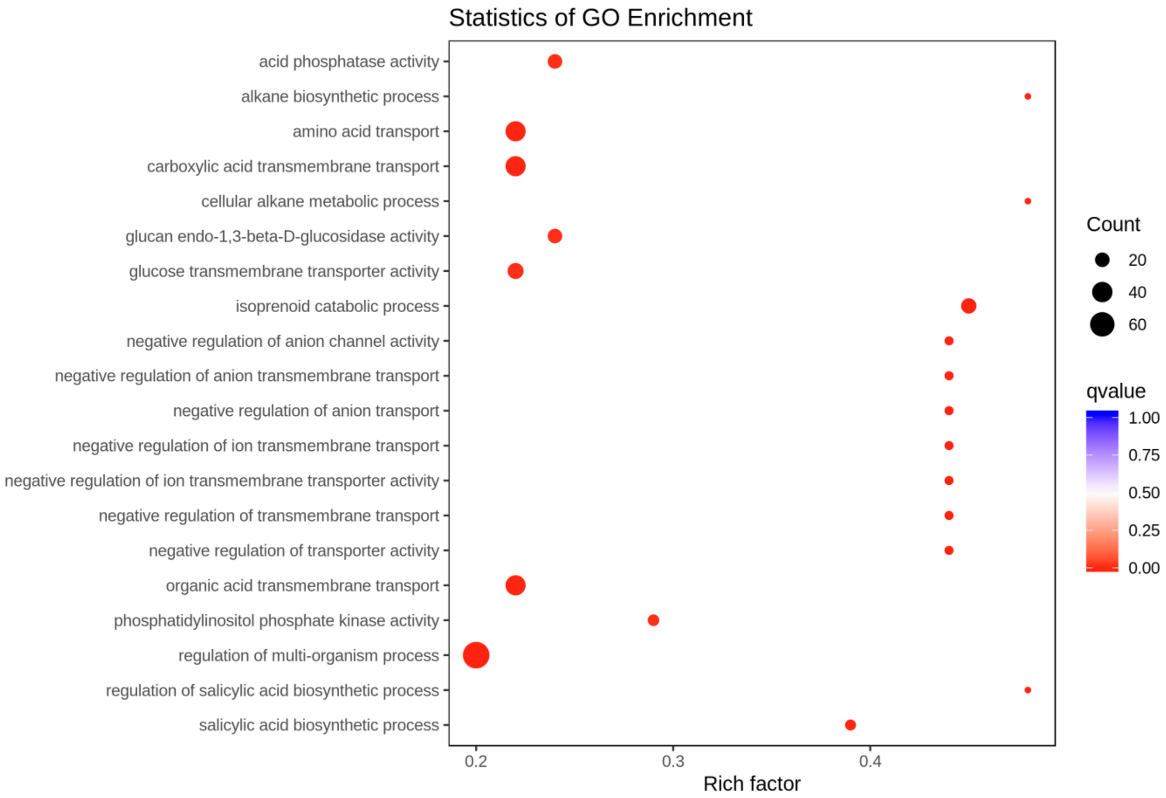

Supplemental Figure S26. GO enrichment scatter-plots of DEGs analysis between the control group versus salinity combined with waterlogging. Y axis indicates the KEGG pathway and X axis the rich factor the larger the rich factor, the greater the enrichment, the smaller the P value, and the more significant the enrichment.

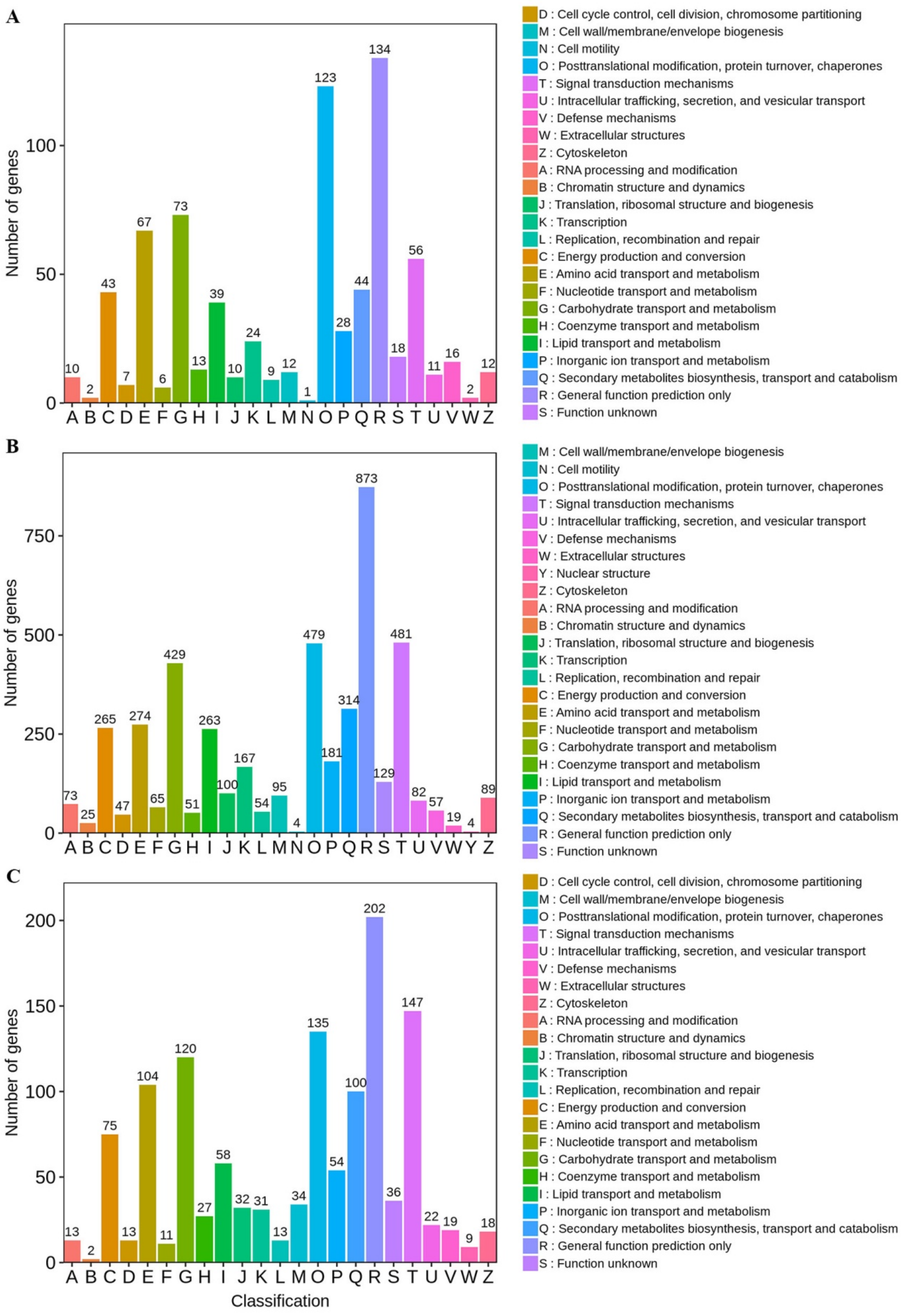

Supplemental Figure S27. Histogram of KOG classification. Control vs salinity (**A**), control vs salinity combined with waterlogging (**B**) and control vs waterlogging (**C**). X axis indicates the functional classification and Y the vertical the number of DEGs.

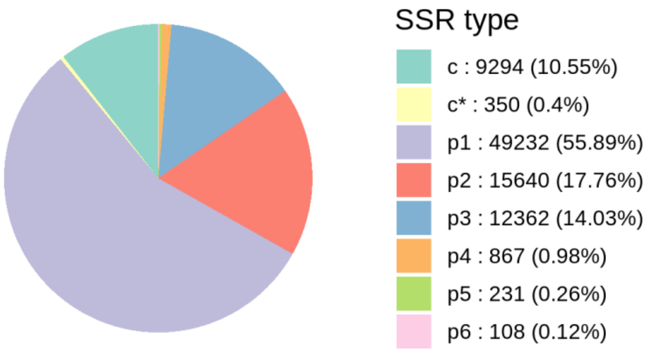

Supplemental Figure S28. A pie chart of the statistical analysis of the number of different types of SSRs

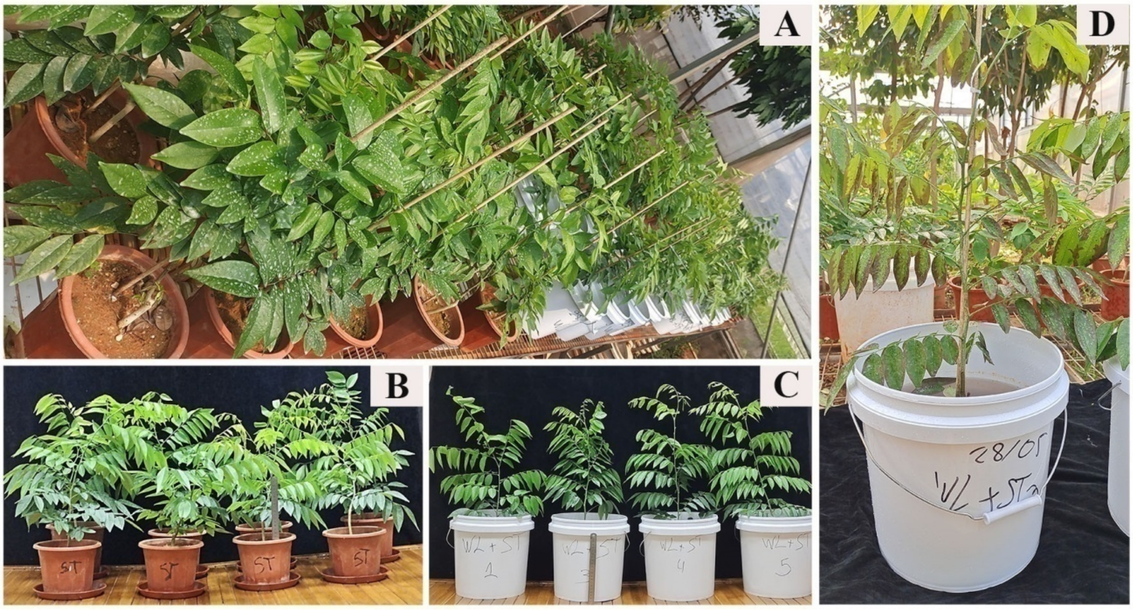

Supplemental Figure S29. Photographs of fragrant rosewood seedlings. Photographs of seedlings arranged in the first experimental table (4 experimental tables in total) at the greenhouse (**A**), group of ST-treated seedlings (**B**) and SWL-treated (100mM of NaCl) seedlings (C) brought in laboratory for indexes measurements (**C**) and seedlings subjected to SWL3 (200mM) affected by chlorosis and necrosis at day 6 (**D**).
